# South African HIV-1 subtype C transmitted variants with a specific V2 motif show higher dependence on α4β7 for replication

**DOI:** 10.1186/s12977-015-0183-3

**Published:** 2015-06-24

**Authors:** Simone I Richardson, Elin S Gray, Nonhlanhla N Mkhize, Daniel J Sheward, Bronwen E Lambson, Constantinos Kurt Wibmer, Lindi Masson, Lise Werner, Nigel Garrett, Jo-Ann S Passmore, Quarraisha Abdool Karim, Salim S Abdool Karim, Carolyn Williamson, Penny L Moore, Lynn Morris

**Affiliations:** Centre for HIV and STI’s, National Institute for Communicable Diseases, A Division of the National Health Laboratory Service, 1 Modderfontein Road, Sandringham, Johannesburg, 2131 South Africa; Faculty of Health Sciences, University of the Witwatersrand, Johannesburg, South Africa; Divison of Medical Virology, Institute of Infectious Disease and Molecular Medicine, University of Cape Town, Cape Town, South Africa; Centre for the AIDS Programme of Research in South Africa (CAPRISA), University of KwaZulu-Natal, Durban, South Africa; National Health Laboratory Service, Groote Schuur Hospital, Observatory, Cape Town, South Africa; Department of Epidemiology, Columbia University, New York, NY USA; ECU Melanoma Research Foundation, Edith Cowan University (ECU), Perth, WA 6027 Australia

**Keywords:** HIV entry, α4β7, Tripeptide-binding motif, Bacterial vaginosis, Cytokines

## Abstract

**Background:**

The integrin α4β7 mediates the trafficking of immune cells to the gut associated lymphoid tissue (GALT) and is an attachment factor for the HIV gp120 envelope glycoprotein. We developed a viral replication inhibition assay to more clearly evaluate the role of α4β7 in HIV infection and the contribution of viral and host factors.

**Results:**

Replication of 60 HIV-1 subtype C viruses collected over time from 11 individuals in the CAPRISA cohort were partially inhibited by antibodies targeting α4β7. However, dependence on α4β7 for replication varied substantially among viral isolates from different individuals as well as over time in some individuals. Among 8 transmitted/founder (T/F) viruses, α4β7 reactivity was highest for viruses having P/SDI/V tri-peptide binding motifs. Mutation of T/F viruses that had LDI/L motifs to P/SDI/V resulted in greater α4β7 reactivity, whereas mutating P/SDI/V to LDI/L motifs was associated with reduced α4β7 binding. P/SDI/V motifs were more common among South African HIV subtype C viruses (35%) compared to subtype C viruses from other regions of Africa (<8%) and to other subtypes, due in part to a founder effect. In addition, individuals with bacterial vaginosis (BV) and who had higher concentrations of IL-7, IL-8 and IL-1α in the genital tract had T/F viruses with higher α4β7 dependence for replication, suggesting that viruses with P/SDI/V motifs may be preferentially transmitted in the presence of BV in this population.

**Conclusions:**

Collectively, these data suggest a role for α4β7 in HIV infection that is influenced by both viral and host factors including the sequence of the α4β7 binding motif, the cytokine milieu and BV in the genital tract. The higher frequency of P/SDI/V sequences among South African HIV-1 subtype C viruses may have particular significance for the role of α4β7 in this geographical region.

**Electronic supplementary material:**

The online version of this article (doi:10.1186/s12977-015-0183-3) contains supplementary material, which is available to authorized users.

## Background

The primary site of HIV replication following infection is the gut associated lymphoid tissue (GALT) [1, 2]. In order to migrate to the GALT and other tissues, leukocytes engage with adhesion molecules expressed on the surface of vascular endothelial cells. One of these receptors is the integrin α4β7 that binds to monomeric gp120 [3]. Since the genital mucosa does not contain organised immune-inductive sites, it relies on α4β7+ T cells to traffic from other sites including the Peyer’s patches [4]. Therefore, the ability of α4β7+ T cells to home to secondary lymphoid tissues and the GALT [5, 6], coupled with their presence at the site of sexual transmission of HIV [7, 8] and co-expression with multiple HIV susceptibility markers [9], suggests that the initial and most relevant site for the gp120-α4β7 interaction is the genital mucosa.

The natural ligands of α4β7 (MAdCAM-1, VCAM-1 and fibronectin [10]) all bind through structurally homologous binding motifs that comprise three residues with a central aspartic acid; Leu-Asp-Thr (LDT), Ile-Asp-Ser (IDS) and Leu-Asp-Val (LDV), respectively [11]. The principal contact sites for these natural ligands are present on the α4-chain [12]. By blocking α4β7 activity with inhibitory antibodies [13], Arthos et al. showed that gp120 binds to α4β7 in a manner that mimics the natural ligands [3]. The V2 domain of gp120 contains a similar tri-peptide motif at position 179–181 (HXB2 numbering) with the aspartic residue at position 180 being 98% conserved across all HIV isolates [3, 14]. The *ITGA4* gene that encodes the α4 subunit shows no polymorphisms in humans and did not correlate with HIV transmission or disease progression [15]. Nevertheless, there appears to be significant variation in the levels of α4β7 reactivity among viruses from different individuals [3]. This suggests that it is the contact residues in gp120 that influence α4β7 affinity. This is bolstered by data that showed differences in the sequence of the α4β7 tri-peptide motif were linked to the differential dissemination potential of distinct HIV-1 genetic forms in China [16]. Recently, Tassaneetrithep et al. described a tri-peptide sequence just upstream of the α4β7 motif as a determinant of integrin binding [17], suggesting that additional viral properties play a role in reactivity with α4β7.

Although gp120 binds α4β7 this interaction is not essential for viral entry, unlike CD4 and CCR5 [3]. Rather, α4β7 is thought to act as an attachment factor, offering a selective advantage for HIV entry by lowering the entropic barrier that slows the ligation of envelope spikes to CD4 and CCR5 [18]. Thus, the gp120–α4β7 interaction may be particularly important during the earliest stages of HIV infection. CD4+ T cells expressing high levels of α4β7 are more susceptible to HIV-1 infection partly because this subset also expresses high levels of CCR5 [9]. This phenotype extends to sites of initial HIV infection such as blood, rectum, colon and genital mucosa of the female reproductive tract [7–9]. However, other studies have failed to confirm any impact of α4β7 on replication in vitro [19–21]. Despite this controversy, when healthy macaques were treated with an anti-α4β7 mAb (Act-1), they were protected from transmission by low-dose SIVmac251 challenge [22]. This antibody also reduced viremia and proviral DNA in the GALT in a high dose challenge model although it did not extend to protection [23]. In addition, a recent study has shown that the number of α4β7^+^ CD4^+^ T cells at the site of rectal transmission is a risk factor for productive HIV infection in rhesus macaques [24]. Sexually transmitted infections such as HSV-2 have also been shown to increase expression levels of α4β7^+^ and enhance the risk for vaginal SHIV infection [25].

To further clarify the role for α4β7 in HIV infection, we made use of longitudinal viruses from the CAPRISA Acute Infection cohort based in Durban, South Africa, a region with one of the highest HIV incidence rates in the world [26]. We devised an α4β7-inhibition replication assay and tested dependence of the viruses on α4β7 for entry and replication using inhibitory mAbs. Here we show that variation in the α4β7 binding motif influences T/F virus α4β7-dependent replication. Furthermore, the immune environment in the genital mucosa at the time of HIV infection correlated with the transmission of particular binding motifs which are highly prevalent in South African subtype C viruses.

## Results

### α4β7 expressed on 293T cells binds infectious HIV

While monomeric gp120 has been shown to bind α4β7 [3], we sought to determine whether biologically relevant forms of HIV envelope expressed on the viral membrane, also bound the integrin. For this, plasmids encoding human α4 and β7 subunits were co-transfected and expressed in 293T cells (which do not express any HIV receptors) and stained with CD49d (α4)-PE (targeting an epitope on α4 overlapping the region of gp120 binding) and β7-FITC to confirm co-expression (Additional file 1, panel A). These cells were used in a direct binding assay, where HIV bound to α4β7 was detected by p24-FITC (Additional file 1, panels B and C). These cells were also used in a competition binding assay, where a reduction of CD49d (α4)-PE binding was measured relative to α4β7+ cells incubated without virus or the inhibitory mAbs, HP2/1 (anti-α4 mAb) and Act-1 (anti-α4β7 mAb). Two types of infectious viruses were used for these experiments; infectious molecular clones (IMCs) which comprised the entire proviral genome of isolates of interest and infectious envelope clones (IECs) which made use of a common pNL4-3 delta Env backbone co-transfected with different gp160 genes.

**Figure 1:**
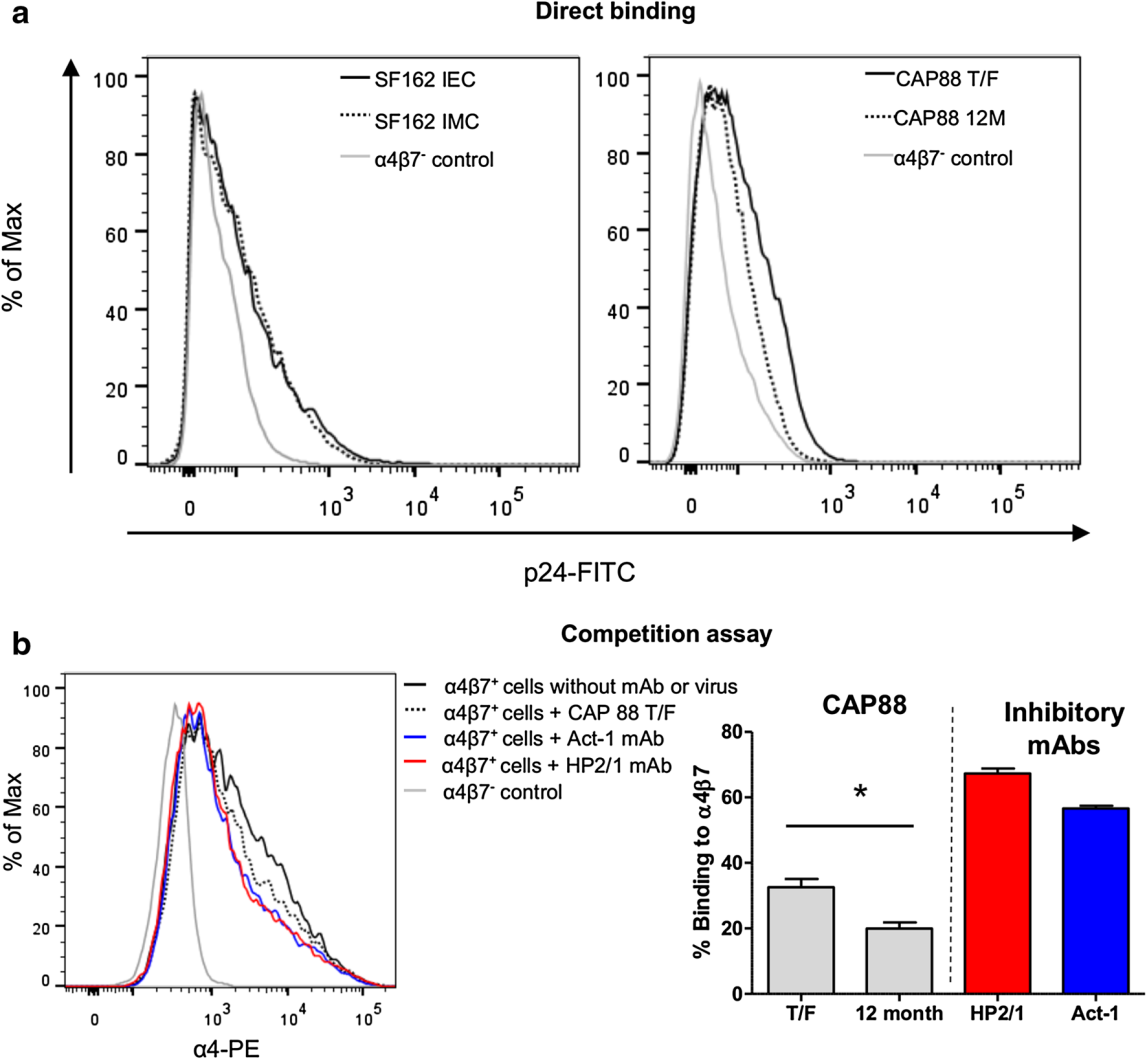
HIV binding to cell-surface expressed α4β7. **a** Direct binding of SF162 (*left panel*) and CAP88 (*right panel*) viruses to α4β7 expressed on 293T cells measured by p24-FITC staining. For SF162, both the IEC and IMC (*solid* and *dashed black lines* respectively) bound α4β7 at similar levels which were higher than the untransfected α4β7^−^ 293T cells (*grey line*). The IEC of CAP88 T/F (*solid black line*) bound better than CAP88 12M (*dashed black line*). This is representative of 3 independent experiments. **b** Competition assay where CAP88 T/F (*black dashed*) bound to the integrin is measured as a decrease in α4-PE antibody binding relative to unbound α4β7 transfected 293T cells (*black solid*). HP2/1 and Act-1 binding in the absence of virus were used as positive controls (*red* and *blue* respectively) while the negative control was untransfected cells incubated with virus. Results are representative of four independent experiments. The CAP88 T/F virus bound significantly better to the α4β7 integrin compared to the 12 month IEC in the competition binding assay as shown in the *bar graph* (p = 0.018; paired t test). *Bars* represent the mean binding percentage of three independent experiments and the error bars represent the SEM.

In agreement with published studies, SF162 was shown to bind to α4β7-expressing cells in a direct binding assay [3, 19] (Figure 1a). No significant differences were noted between the SF162 IMC or IEC in either of the binding assays or between another two IMC/IEC pairs used in this study (CAP210 and CAP239) (Additional file 2). Similar to what was shown previously (using monomeric gp120) [27], the CAP88 T/F IEC bound better to α4β7 expressed on 293T cells than the CAP88 12 month IEC (Figure 1a). These findings were confirmed in a competition assay where the CAP88 T/F was better able to compete for integrin binding with fluorescently labelled α4β7-directed mAbs than the 12 month IEC (*p = 0.018; paired t test), although not as efficiently as the inhibitory mAbs (Figure 1b). These data confirm the capacity of biologically relevant cell surface-expressed HIV-1 Env to bind α4β7.

### Anti-α4β7 antibodies partially inhibit virus replication

We next determined whether binding to α4β7 enhanced HIV infection of primary CD4+ T cells induced to express α4β7 using *all*-*trans* retinoic acid (ATRA), the effect of which was measured by flow cytometry (Additional file 3). For this, we developed an α4β7 inhibition replication assay which measured viral replication (levels of p24) in ATRA-treated CD4+ T cells in the presence of HP2/1 or Act-1 mAbs over a 10 days period. Accurate titration of the inhibitory mAbs was crucial as saturating concentrations were shown to enhance viral replication in this assay (Additional file 4). This effect is likely to be the result of signalling processes and homotypic clustering of cells enhanced by ligands to and antibodies against α4β7 [12, 19, 28]. This titration is unique to this study and may have allowed us to develop an assay in which we see consistent partial inhibition of replication by the inhibitory mAbs, in contrast to other studies [19, 21]. The optimal mAb concentration for viral inhibition by HP2/1 was 0.275 nM and for Act-1 was 2.2 pM which were used in all subsequent experiments.

**Figure 2:**
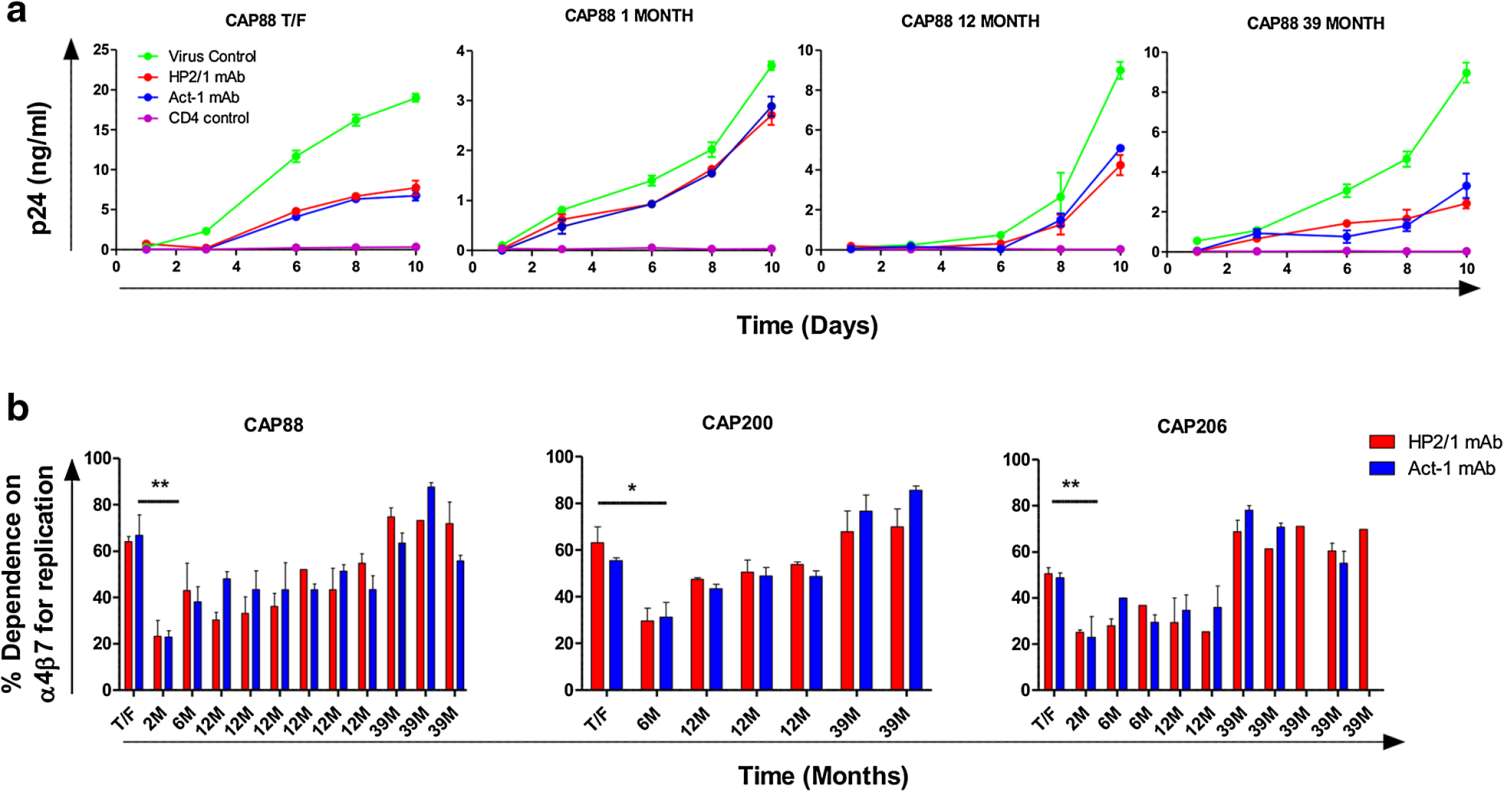
HIV dependence on α4β7 for replication over the course of infection. **a** Kinetic growth curves of IECs from CAP88 T/F, 1 month, 12 and 39 months post-infection (*green lines*) in the presence of α4β7 inhibitory mAbs Act-1 (*blue*) and HP2/1 (*red*) or anti-CD4 mAb (*purple*) measured as p24 (ng/ml) over 10 days. Inhibition by Act-1 and HP2/1 was partial at the point of exponential growth of the virus control and did not differ between the two mAbs. *Curves* are representative of three independent experiments, each one in triplicate and *error bars* representative of SEM. **b** Longitudinal IECs (from 3 individuals) including the T/F virus and multiple 2–39 months post-infection viruses were tested for their dependence on α4β7 for viral replication. The percentage inhibition by HP2/1 (*red*) and Act-1 (*blue*) is expressed as the difference in p24 concentration between HP2/1 or Act-1 treated and untreated cells at the point of exponential viral growth. Differences in dependence across all time points in each of these three individuals were significant by a repeated measures ANOVA (p < 0.0001) as well as between CAP88 T/F and 1 month p.i, (**p < 0.001) CAP200 T/F and 6 months p.i. (*p < 0.01) and CAP206 T/F and 2 months p.i (**p < 0.001). Pairwise comparisons were adjusted by the Tukey method. *Bars* represent means of between two and three independent experiments with the *error bars* indicating SEM.

A total of 60 IECs from 11 CAPRISA participants were tested in this assay to determine if the α4β7 receptor was commonly used by HIV. While replicative capacity differed among IECs, all showed lower levels of replication (range 15–88%) in the presence of antibodies to α4β7, confirming usage of the integrin (Additional file 5). There was no difference in replication inhibition levels between HP2/1 and Act-1, suggesting that the inhibition was a result of blocking the heterodimeric α4β7 surface molecule (Figure 2a). Treatment with anti-α4β7 mAbs resulted in only partial inhibition of replication, compared to an anti-CD4 mAb that resulted in complete abrogation of replication of all viruses consistent with its essential role in viral entry. These data re-enforce previous findings that α4β7 serves as an attachment factor and unlike CD4 and CCR5, is not essential for HIV infection [3, 19, 21, 29].

### The dependence on α4β7 for viral replication changes over time

In order to assess whether the dependence on α4β7 varied over the course of HIV infection, we selected three participants who had a complete longitudinal set of IECs, including the T/F virus and viruses from shortly after infection (1 or 2 months post-infection) as well as later time points up to 3 years post-infection. All 3 individuals showed a similar pattern, with the T/F virus having a greater dependence on α4β7 for replication than viruses from 1 to 2 months (CAP88, CAP200 and CAP206; p < 0.001, p < 0.01 and p < 0.001 respectively using a repeated measures ANOVA) (Figure 2b). However, α4β7 dependence of viruses from later time points was not significantly different from T/F viruses. Longitudinal samples from additional CAPRISA participants showed a similar trend, but this analysis was limited because clones from either the T/F or later time-points were not available (Additional file 5). We found no association between dependence on α4β7 and markers of disease progression such as CD4 counts or viral loads when corrected for duration of infection (data not shown).

Analysis of longitudinal sequences revealed no changes in the α4β7 tri-peptide motif in CAP88 and CAP206 while in CAP200 there was a change from SDV to PDI by 6 months post infection, but this was due to dual infection (Sheward, unpublished) (Additional file 6). For CAP88, there were only three amino acid differences between the T/F and the 2 month clone; L568R, a highly conserved residue in the N-heptad repeat of gp41 and two changes in the cytoplasmic tail. For the CAP206 pair there was an introduction of a predicted N-linked glycan (PNG) at position 462 in V5 and a D474N mutation in the C5 region (Additional file 7). CAP200 showed a total of 39 non-synonymous changes in envelope at 6 months Overall, no common sequence signature was associated with changes in α4β7 reactivity among these three participants.

Since decreased loop length and PNG density of the gp120 V1/V2 and C3/V4 regions have previously been shown to correlate with increased α4β7 binding [27], we analysed these features among all 60 clones. We found that α4β7 dependence positively correlated with the length and predicted glycan density of V1/V2 which includes the α4β7 binding site and negatively correlated with C3/V4 length, however these associations were only weakly supported (Additional file 8, panel A). We also compared individual predicted glycan sites (excluding those in variable regions which could not be accurately aligned) across all 60 sequences (Additional file 8, panel B). Viruses with high α4β7 dependence had significantly higher frequencies of PNG234 (p = 0.009) and PNG334 (p = 0.006), while PNG332 (p = 0.026) was present less frequently as compared by the Fisher exact test. These data suggest that specific glycans may play a role in α4β7 dependence but it is likely that additional factors influence interactions with the integrin.

### Impact of host factors at transmission on α4β7 dependence for replication

**Figure 3:**
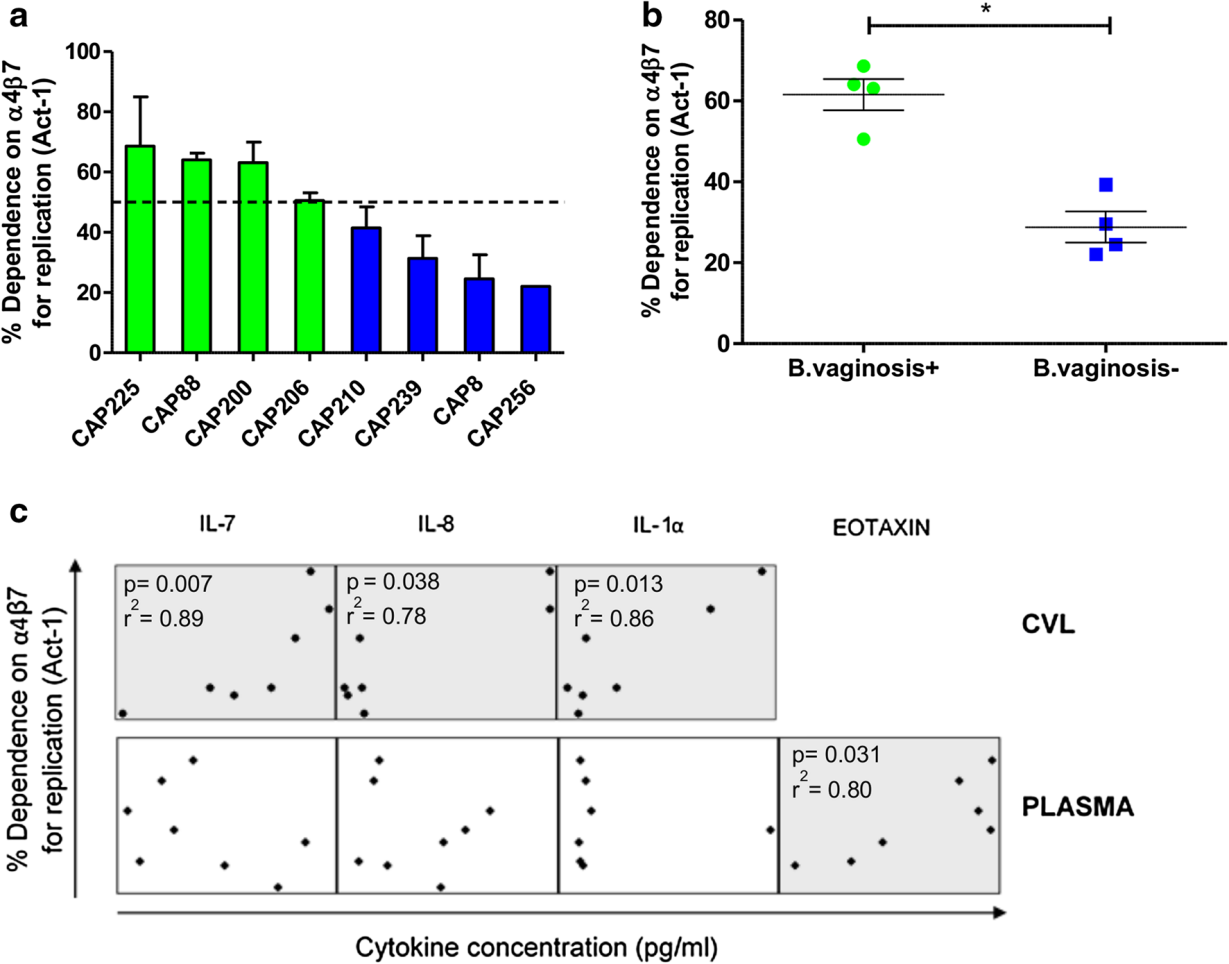
Bacterial vaginosis and genital cytokines associated with α4β7-dependent T/F viruses. **a** T/F viruses of CAP225, CAP88, CAP200 and CAP206 showed high (>50%, indicated by the *dotted line*) α4β7 dependence (*green*) while the remaining four showed lower α4β7 dependence (*blue*). Dependence on α4β7 was determined using Act-1 mAb inhibition. *Bars* represent the mean of four independent experiments, with *error bars* indicating SEM. **b** Individuals infected with T/F viruses that had higher dependence on α4β7 were significantly more likely to be BV positive at the time of infection (p = 0.029; Mann–Whitney test). **c** Concentrations of cytokines in CVL (n = 31) and plasma (n = 30) were determined and correlated with T/F virus dependence on α4β7 for replication. IL-7, IL-8 and IL-1α showed significant univariate correlations in the CVL. In contrast only eotaxin was significantly associated in plasma (shown in the *grey boxes*) while IL-8, IL-1α and IL-7 showed no correlation in plasma. No significance was maintained at a multivariate level. Relevant p values and Spearman’s coefficients are shown where *p < 0.05.

Since the role of α4β7 is likely to be most relevant at transmission, we focused on the 8 T/F viruses included in this study. These exhibited a wide range of Act-1 inhibition (22–69%) indicating that high α4β7 dependence is not a typical feature of T/F viruses (Figure 3a). We investigated if this variation could be explained by host factors present at the time of transmission. STIs have been identified as a major cause of inflammatory cytokine upregulation and immune cell recruitment to the genital mucosa, in some cases typified by the homing function of α4β7 [7, 30]. Given this and the integrin’s role in supporting HIV replication, we considered whether these factors could create an environment conducive for α4β7 interaction. Only *Trichomonas vaginalis*, *Chlamydia trachomatis* and bacterial vaginosis (BV) were detected in some individuals at the time of transmission. Strikingly, individuals with BV (Nugent scores ≥7) had viruses with significantly higher dependence on α4β7 than those who did not (61.58 vs. 29.64% α4β7 dependence; p = 0.029 Mann–Whitney test) (Figure 3b).

Next, we investigated the cytokine milieu in the genital compartment at the time point when the T/F viruses were isolated. The concentrations of interleukin (IL)-7 (r^2^ = 0.89), IL-8 (r^2^ = 0.78) and IL-1α (r^2^ = 0.86) in cervicovaginal lavages (CVLs) correlated significantly with α4β7 dependence in a univariate analysis (p = 0.007, p = 0.038, p = 0.013 respectively by Spearman’s correlation) but this was lost when the p-values were adjusted for multiple comparisons, likely a result of the small sample size (Figure 3c; Additional file 9). These associations were not mirrored in plasma where only eotaxin, an eosinophil chemoattractant whose function is mediated by α4β7 [31], showed a significant univariate correlation (p = 0.031; r^2^ = 0.80) but a non-significant adjusted p value of 0.899.

### The sequence of the α4β7-binding motif influences virus binding and replication

**Figure 4:**
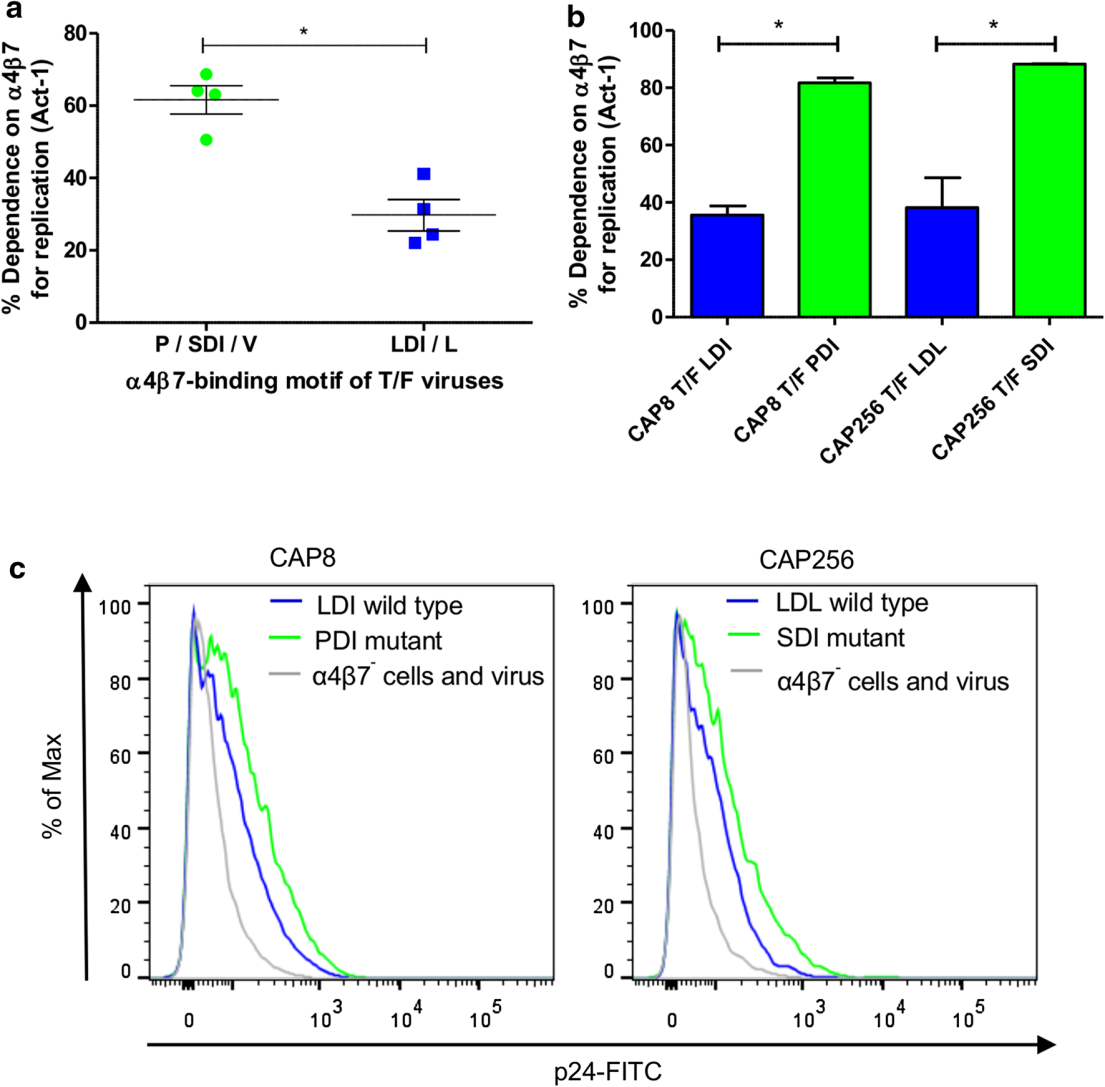
Viruses with P/SDI/V α4β7 binding motifs are more reactive with the integrin. **a** T/F viruses with P/SDI/V showed a higher dependence on the integrin compared to those with LDI/L motifs (n = 4, *blue*) (*p = 0.029; Mann–Whitney test). **b** Mutation of CAP8 T/F from LDI to PDI and CAP256 T/F from LDL to SDI resulted in a significant increase in α4β7 dependence (*p = 0.02 for both, paired t test). *Bars* represent three independent experiments and *error bars* indicate the SEM. **c** CAP8 and CAP256 mutants also showed increased α4β7 binding compared to the wild-type in the direct assay using p24-FITC MFI as a read-out, representative of three independent experiments.

While the aspartic acid in the tri-peptide binding motif in the V2 domain is highly conserved, there is variation at the first and third amino acid residues. Interestingly, the sequence of the tri-peptide α4β7 binding motifs could be used to stratify the 8 T/F viruses based on replication dependence (Figure 4a). The 4 T/F viruses with high α4β7 reactivity had P/SDI/V motifs while the 4 T/F viruses with low α4β7 reactivity had LDI/L motifs (p = 0.029, Mann–Whitney test).

To determine if the sequence of the motif impacted on binding to α4β7, we mutated the tri-peptide motif from P/SDI/V to LDI/L (or vice versa) in 5 of the 8 T/F viruses. The α4β7 binding motif of the CAP8 T/F virus (which showed low α4β7 dependence) was mutated from LDI to PDI. Similarly the CAP256 T/F was mutated from LDL to SDI. In both cases, mutated viruses had significantly increased α4β7 dependence (p = 0.02, paired t test, Figure 4b). Furthermore, binding to the integrin as measured by the direct p24 binding assay was enhanced when motifs were mutated from LDI/L to P/SDI (Figure 4c). Notably, when viral motifs were mutated from P/SDI to LDI/L, none of the mutated viruses were able to replicate.

### Analysis of the α4β7 binding motif, presence of BV and the genital and plasma cytokine milieu in the CAPRISA 002 cohort

**Figure 5:**
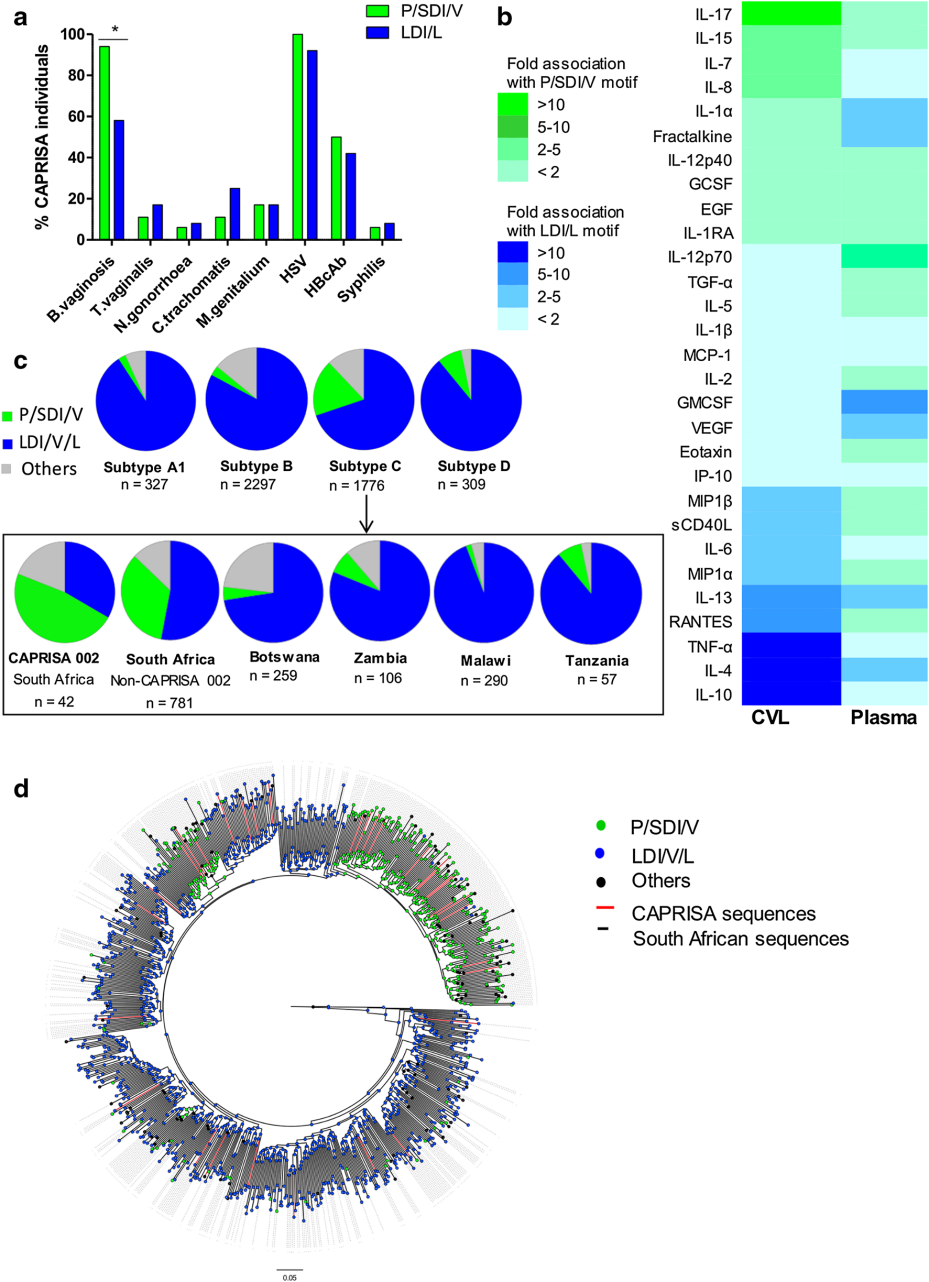
Viruses with P/SDI/V motifs are associated with BV and are more frequent among South Africa subtype C viruses. **a** P/SDI/V α4β7 binding motifs were significantly associated with concurrent BV infection as compared to those with LDI/L motifs (17/18 P/SDI/V motifs vs. 7/12 LDI/L motifs; *p = 0.026, Fisher exact test). **b** Fold differences of mean cytokine levels in CVL and plasma among 25 and 28 individuals respectively in the CAPRISA 002 cohort separated based on those that have P/SDI/V motifs (*green*, n = 15 and n = 17) and those that have LDI/L motifs (*blue*, n = 10 and n = 11). The intensity of the respective colours is indicative of the fold differences between the two groups (*green* = greater fold difference in P/SDI/V group; *blue* = greater fold difference in LDI/L group) which is ranked based on the CVL profile. **c** Global frequency of the P/SDI/V motif (*green*), LDI/V/L motif (*blue*) and other motifs (*grey*) among sequences from subtypes A, B, C and D from the Los Alamos database. Further breakdown of subtype C sequences according to the country of origin is shown in the *box*. Viruses from South Africa showed the highest frequency of P/SDI/V motifs (35%) with those from the CAPRISA 002 cohort exceeding this (48%; n = 20). **d** A maximum likelihood tree inferred using Fasttree of all subtype C gp160 sequences from the LANL database (n = 776) rooted on the 1959 Zaire sequence with HXB2 as an outgroup. Nodes are coloured according to the α4β7 binding motif (positions 179–181), South African sequences indicated by *dotted lines* and CAPRISA sequences indicated by *red lines*.

To further examine the host and viral factors associated with α4β7 dependence, we analysed additional women in the CAPRISA 002 cohort where STI, cytokine and sequence data were available. Of the 30 women who had STI clinical information, 18 were infected with viruses with P/SDI/V and 12 with LDI/L motifs. In this larger analysis, BV diagnosis (Nugent score ≥7) was significantly associated with viruses having P/SDI/V motifs (17/18 P/SDI/V motifs vs 7/12 LDI/L motifs; p = 0.026, Fisher exact test) (Figure 5a), mirroring what was seen in the smaller sub-group of 8 women. Using available cytokine concentrations for CVL (n = 25 women) and plasma (n = 28 women), we investigated the relationship with different viral motifs in each compartment. CVL exhibited a dramatically different association profile to plasma (Figure 5b). In CVL, IL-17 showed the strongest positive association with the P/SDI/V motif and IL-10 the strongest inverse correlation, although these did not correlate with α4β7 dependence (Additional file 9). Of the three cytokines in CVL (IL-7, IL-8 and IL-1α) that were previously shown to correlate with α4β7 dependence for replication (Figure 3c), IL-7 and IL-8 concentrations were 2- to 5-fold higher in CVL from women who were infected with viruses containing P/SDI/V motifs compared to women without these motifs in agreement with the original observation.

### South African HIV-1 subtype C viruses show a higher frequency of P/SDI/V motifs

Among the 42 women where viral sequence data was available, 20 (48%) were infected with viruses that contained P/SDI/V motifs, exceeding those with LDI/V/L motifs (n = 14; 33%) (Figure 5c). To explore whether this was typical of global viruses, we analysed ~4,700 unique V1/V2 sequences from the Los Alamos National Laboratory (LANL) HIV database with information on genetic subtype, route of transmission, sex of individual and region. HIV-1 subtype C showed the highest prevalence of viruses with P/SDI/V motifs (18%), in contrast to subtypes A, B and D (2, 3 and 8% P/SDI/V motifs respectively). When subtype C sequences were broken down by region, however, the increased frequency of the P/SDI/V motif was almost completely accounted for by South African viruses. Of 781 sequences from South Africa, excluding sequences from the CAPRISA 002 cohort, 273 (35%) had the P/SDI/V motif. This is in contrast to other regions where subtype C circulates, including Tanzania, Malawi, Botswana and Zambia where the frequency of the P/SDI/V motif was less than 8%. Since many South African sequences were from women and the route of transmission is predominantly heterosexual, we compared gender and transmission route across all sequences in the database. However, no significant differences were noted between the groups (not shown).

When the phylogeny of full-length subtype C gp160 sequences (n = 776) was examined the enrichment of non-LDI/L motifs in South African sequences (indicated by the dotted lines) was found to be due to founder effects (Figure 5d). Two distinct South African lineages with founders that had PDI motifs with branch support of 0.955 and 0.999 were identified. These data suggest that the high frequency of P/SDI/V motifs is not typical of subtype C viruses in general, but rather represents a signature of South African subtype C viruses.

## Discussion

While it is generally accepted that gp120 binds α4β7 via a tri-peptide motif in V2, its role in HIV infection is controversial [19–21, 27, 29]. Using viruses from the well-characterized CAPRISA acute infection cohort, we were able to show that interaction with α4β7 was common but highly variable between isolates from different individuals as well as within an individual over time. Furthermore, T/F viruses with a P/SDI/V tri-peptide motif bound α4β7 more efficiently and had higher dependence on α4β7 for replication compared to those with an LDI/V motif. Notably, the P/SDI/V genotype was more common among South African subtype C viruses. The disproportionate distribution of P/SDI/V genotypes in the South African-based CAPRISA cohort may in part explain why this study has enabled us to more clearly define a role for α4β7 in HIV infection.

Our study used infectious viruses from the same individuals over multiple time points, and to our knowledge, includes the largest collection of T/F viruses investigated for α4β7 dependence linked to extensive clinical and laboratory data. For those individuals where we had complete longitudinal datasets (CAP88, CAP200 and CAP206), we found α4β7 dependence was high for the T/F viruses but decreased during acute infection. However, high dependence on α4β7 was not a feature common to all T/F viruses and is therefore unlikely to be a transmission signature. The loss of high reactivity also coincides with migration to the target tissue after which α4β7 would no longer provide any selective advantage [32]. Many of the chronic viruses tested also had high α4β7 dependence but its role at this stage of infection is less clear. Comparison of the longitudinal sequences from these 3 participants revealed no changes in the α4β7 binding motif except for CAP200, who was dually-infected. However, we noted changes in other regions such as V5 and gp41. Glycan changes in V5, that forms part of the conformational epitope for CD4 binding mAbs such as VRC01 [33] may affect α4β7 reactivity as the integrin is in close proximity to CD4 on the cell surface [9, 34]. Similarly, changes in gp41 may impact on membrane fusion and viral incorporation, with one of the changes, L568R, associated with a reduction in the ability of HIV-1 envelope protein expressing cells to fuse with target cells [34]. Regardless, the absence of common changes between the T/F and acute/early viruses in V1/V2 suggests that regions outside this domain influence α4β7 reactivity. We did find that α4β7 dependence was weakly associated with higher glycan density and longer V1/V2 loops. This is at odds with Nawaz et al. [27], although both studies found that shorter C3/V4 domain lengths was associated with increased α4β7 binding affinity. In addition to this, we found the presence or absence of specific glycans correlated with α4β7 reactivity. The under-representation of PNG332 in viruses with high α4β7 dependence and the reciprocal overrepresentation of PNG334 is intriguing as the 332 glycan has been found to occur less frequently in T/F viruses [35]. These findings suggest that while differences in predicted glycan occupancy alone cannot explain α4β7 reactivity, their position may impact the ability of the tri-peptide motif to access α4β7 on the cell surface. Recent data suggests that the tri-peptide motif is occluded in the pre-fusion trimer structure [36], however this crystalline structure does not account for the significant flexibility and entropy associated with the variable domains of gp120 in which transient exposure of the α4β7 binding site is plausible. Non-functional trimers on the surface of HIV could also provide additional opportunities for viral particles to bind α4β7 [37].

STIs have been identified as a major cause of inflammatory cytokine upregulation and immune cell recruitment to the genital mucosa [38] increasing HIV risk [39–41]. Both HSV-2 and *Chlamydia trachomatis* infection increase the number of α4β7+ CD4+ T cells in the genital tissue [7, 30, 42]. Bacterial vaginosis, a common syndrome characterised by a shift in vaginal flora composition [43], has been identified as a specific risk factor for HIV acquisition, also influencing genital tract cytokine concentrations [40, 44, 45]. In this study, we found that BV diagnosis at the time of HIV infection was associated with T/F viruses that had high α4β7 dependence for replication, but found no such associations with STI’s. In a recent study by Masson et al., BV in the CAPRISA cohort upregulated inflammatory cytokines while downregulating chemokines in the CVL, in contrast to chlamydia and HSV-2 [46]. Chemokines are essential for the chemotaxis of effector cells, suggesting that BV may be associated with a reduction in target cells in the genital mucosa, favouring viruses with enhanced α4β7 reactivity. However, since the data presented here does not prove causality, it is possible that the increased α4β7 dependence is merely a feature of viruses transmitted in these conditions. This is corroborated by our observation that individuals infected with viruses with P/SDI/V motifs were more likely to have BV at the time of infection. Overall, BV-associated changes in the genital cytokine milieu may modulate the mucosal barrier favouring the transmission of viruses with P/SDI/V α4β7 binding motifs that have a high dependence on the integrin.

We also found that IL-7 levels in genital secretions at transmission were associated with α4β7 dependence of T/F viruses. Recently, Cimbro et al. demonstrated that IL-7 induces expression and functional activation of α4β7 in vitro and in vivo [47]. The levels of IL-7 required to upregulate the integrin, however, are associated with lymphopenia; an immunological state only generated late in HIV infection. It is therefore unlikely that the levels induced in the CVL at the time of infection would be sufficient to influence α4β7 expression. Interestingly, other cytokines upregulated by BV, such as IL-8 and IL-1α [48–51] were also associated with α4β7 dependence in CVL, but not in plasma, suggesting that that the most relevant impact of the cytokines on α4β7 is at the site of transmission. Furthermore, all 3 cytokines were associated with the P/SDI/V α4β7 binding motif, further suggesting that BV may play a significant role in determining the level of α4β7 interaction at transmission.

This study is the first to fully explore the effect of different α4β7 sequence binding motifs on α4β7 reactivity. Transmitted viruses with P/SDI/V motifs, present in only 8% of global viruses, were associated with a higher dependence on α4β7 for replication and bound better to the integrin. LDI/L/V motifs which most closely resemble those of the natural ligand MAdCAM-1 (LDT) and fibronectin (LDV) are more common in global sequences accounting for 78% of all circulating variants. In addition to the LDT motif, the integrin-binding loop of MAdCAM-1 contains a downstream SDT motif that is responsible for stabilisation of the interaction [52]. Interestingly, an α4β7 antagonist containing the SDV/T motif was more potent than one containing an LDT motif [53]. This suggests that different motifs display different affinities for the ligand, similar to the observation in this study that viruses with P/SDI/V motifs have a higher reactivity with α4β7 as compared to LDI/V motifs.

Unexpectedly, we found that 48% of women in the CAPRISA 002 cohort and 35% of all South African sequences had P/SDI/V motifs, significantly higher than what is seen in other countries where HIV-1 subtype C circulates. This is due in part to founder effects, with two South African lineages having founder viruses with a PDI motif. The introduction and maintenance of this motif in South African viruses may be as a result of an immunological feature of people in this region. α4β7 expression is known to be regulated by ATRA, a metabolite of vitamin A [54]. Vitamin A deficiency is common in the South African population as compared to other sub-Saharan African countries; with 19% of pregnant women having serum retinol <0.70 µmol/l, higher than Botswana, Malawi or Tanzania [55]. It is therefore possible that vitamin A deficiency may result in decreased expression of α4β7, leading to the selection of viruses with a α4β7 binding motifs that have a greater affinity for the integrin in this population. In addition, the exact composition of the microbiota in BV has not been fully elucidated, and it is possible that this may differ from region to region; possibly creating a unique environment in the South African population. This data highlights the importance of fully characterising individual epidemics, even those caused by the same subtype.

## Conclusions

Collectively, these data suggest a role for α4β7 in HIV infection that changes over time and that is impacted during transmission by a number of factors including the sequence of the α4β7 binding motif, cytokine milieu and BV in the genital tract. This may have particular relevance for South Africa where a higher frequency of α4β7-dependent V2 motifs were present among HIV-1 subtype C transmitted/founder viruses. Further understanding of the interaction between the virus and the integrin might provide additional opportunities for devising vaccine and therapeutic strategies.

## Methods

### Ethics statement

Ethical approval was received for the CAPRISA 002 Acute Infection Study from the University of KwaZulu-Natal, University of Cape Town and University of Witwatersrand. All participants provided written informed consent for the CAPRISA 002 study with tacit agreement to adhere to regular clinic visits and blood draws. Approval for this particular study was granted by the University of the Witwatersrand.

### Study participants

Participants in the CAPRISA 002 Acute Infection cohort, established in 2004 [26] were derived from prospective cohorts of high-risk HIV negative South African women [46]. Clinical and laboratory monitoring including CD4 count, viral load and testing for STIs and BV were performed routinely [26, 40] on all participants at enrolment and regularly thereafter. Eleven participants, where cloned longitudinal envelopes from the transmitted/founder and/or acute infections were available, were selected for the α4β7 binding and replication studies. Participants in the parent CAPRISA 002 cohort with sequence (n = 42), laboratory-diagnosed STIs or BV (n = 28) and cytokine (n = 25) data were available for a larger correlates study.

### Testing for STIs and BV

A gynaecological examination was performed and two vulvovaginal swabs were collected [40]. Swabs were screened for *C. trachomatis*, *N. gonorrhoeae*, *M. genitalium*, HSV and *T. vaginalis* by PCR. BV was diagnosed by Gram staining using Nugent’s criteria (a score ≥7 indicates BV).

### Cell lines

The TZM-bl cell line engineered from CXCR4-positive HeLa cells to express CD4, CCR5, and a firefly luciferase reporter gene (under control of the HIV-1 LTR) was obtained from the NIH AIDS Research and Reference Reagent Program, Division of AIDS, NIAID, NIH (developed by Dr. John C. Kappes, and Dr. Xiaoyun Wu [56, 57]). The 293T cell line was obtained from Dr George Shaw (University of Alabama, Birmingham, AL, USA). Cell lines were cultured at 37°C, 5% CO_2_ in DMEM containing 10% heat-inactivated fetal bovine serum (Gibco BRL Life Technologies) with 50 ug/ml gentamicin (Sigma) and disrupted at confluency by treatment with 0.25% trypsin in 1 mM EDTA (Sigma).

### Isolation and stimulation of CD4+ T lymphocytes from whole blood

CD4+ T cells were isolated to a purity of 99% from healthy blood donors using RosetteSep Human CD4+ T cell Enrichment Cocktail (Stem Cell Technologies, Canada) as per the manufacturer’s protocol. Isolated cells were cultured with 10 µM *all*-*trans* retinoic acid (ATRA) (Sigma-Aldrich, MO, USA), 50 ng/ml OKT3 (anti-CD3 antibody) (eBioscience, CA, USA) and 20 U/ml IL-2 (Roche Applied Sciences, Germany) in RPMI media supplemented with 20% heat inactivated fetal bovine serum for 6 days at 37°C, 5% CO_2_ [3]. The purity of the CD4+ T cell population as well as the upregulation of α4β7 by ATRA was confirmed routinely by flow cytometry. Donors were designated as responders or non-responders based on whether ATRA treatment upregulated the α4β7+ population of lymphocytes as shown in Additional file 3. Only 25% of donors responded to ATRA treatment similar to what was shown in a study by Arthos et al. [3] and only these donors were used in further experiments.

### Flow cytometry surface staining

Isolated CD4+ T cells from ATRA-treated responders were stained with optimally titrated fluorescently labelled antibodies: CD3 energy coupled dye (ECD Beckham Coulter, France), CD4-Quantum dot 605 (Invitrogen, Carlsbad, CA, USA), anti-human CD49d (Integrin alpha 4) R-Phycoerythrin (PE) (eBioscience, CA, USA), anti-human/mouse integrin β7 fluorescein isothiocyanate (FITC) (eBioscience, CA, USA) and Aqua Fluorescent Reactive Dye (Invitrogen, Molecular Probes, Carlsbad) in 1% BSA/PBS staining buffer in the dark for 30 min at 4°C and fixed in 0.1% paraformaldehyde/PBS. Fluorescence minus one controls were used to define the gating strategy and compensation was done using anti-mouse Igκ BD CompBeads. Acquisition of all samples was performed on a FACSAria (BD Biosciences) and analysis done on FlowJo X software (TreeStar Ashland, OR, USA).

### Production of infectious viruses and pseudoviruses

Cloned HIV-1 gp160 envelope genes derived by single genome amplification as previously described [58] from selected CAPRISA participants were used to produce infectious virions. Mutant envelopes were generated as detailed in Table 1 with QuikChange Lightning Kit (Stratagene) and confirmed by DNA sequencing. Infectious envelope clones (IECs), were generated by re-amplifying the gp160 *env* gene from the envelope plasmids and co-transfecting the PCR product with the pHIVΔenvBstEllnef-hisD backbone (a gift from Dr Daniel Kuritzkes) which were constructed as described elsewhere [59]. Two infectious molecular clones (IMCs) which contain full proviral genomes derived from participants CAP210 and CAP239 and representative of the T/F viruses were also used. The SF162 IMC was from David Montefiori at Duke University, NC. The IMC plasmids were transfected in 293T cells and both IECs and IMCs were incubated at 37°C, 5% CO_2_ for 72 h following which the media was changed to fresh DMEM with 10% FBS. After a week of incubation in 293T cells, 1 ml of the viral supernatant was spinoculated for 60 min at 1,200 g, 30°C with activated CD8+ depleted PBMCs at 5 × 10^6^ cells/ml and the spinoculation was repeated the next day. Viral growth was monitored by an in-house p24 antigen ELISA as described previously [60]. p24 positive cultures were expanded into T25 flasks with fresh CD8 depleted PBMCs and following a week of incubation, the viral supernatant was removed, filtered with a 0.2 nm micropore filter and aliquoted for freezing at −70°C. All viruses inputs were titrated to a TCID_50_/ml of 25 as described elsewhere [56, 57].

**Table 1:** Mutations introduced into the α4β7 binding motif of T/F viruses

Individual	Type	α4β7 binding motif^a^	IEC
CAP225 T/F	Wild type	SDI	✓
	Mutant	*L*DI	Did not grow
CAP88 T/F	Wild type	PDI	✓
	Mutant	*L*DI	Did not grow
CAP200 T/F	Wild type	SDV	✓
	Mutant	*L*D*I*	Did not grow
CAP8 T/F	Wild type	LDI	✓
	Mutant	*P*DI	✓
CAP256 T/F	Wild type	LDL	✓
	Mutant	*S*D*I*	✓

^a^Introduced mutations are in italics.

### Sequence analysis

Full length *env* sequences from the CAPRISA 002 cohort were assembled and edited using Sequencher v.4.5 and Bioedit v.7.0.5.3. Number and positions of predicted N-linked glycans were estimated using the N-GlycoSite tool [61]. Variable domain lengths and relative positions of predicted glycans were determined with reference to HXB2 numbering. For relative frequency of predicted glycan sites, envelopes that showed α4β7 reactivity above the mean were defined as having high α4β7 dependence (n = 33) and those with values below the mean were considered to have low α4β7 dependence (n = 27). The frequency of predicted glycans (excluding those in the variable regions that are difficult to align accurately) were expressed as a proportion of either high or low α4β7 dependent sequences. V1/V2 sequences downloaded from Los Alamos National Laboratory HIV database were selected as single unique sequences per individual. These sequences were further stratified by country of sampling, genetic subtype, mode of transmission and gender of infected individual. Phylogeny of gp160 of subtype C viruses (n = 776) was assessed using the Shimodaira–Hasegawa test and Fasttree to determine a maximum-likelihood tree and branch support.

### Flow cytometry based α4β7-virus binding assays

α4 and β7 encoding plasmids synthesised by Origene (Rockville, MD, USA) were co-transfected using 4 µg of each plasmid into 293T cells with X-tremeGENE Transfection Reagent (Roche) or Polyethyleimine Max (Polysciences, Inc., Warrington, PA, USA) and incubated at 37°C, 5% CO_2_ for 2 days. The transfected cells were removed gently with 1 mM EDTA/PBS, following which the co-expression of α4β7 was determined by flow cytometric staining using CD49d (integrin α4)-PE (eBioscience, CA, USA) and anti-human/mouse integrin β7 FITC as described above. The gating strategy was single (FSC-H/FSC-A), live (FSC/Aqua vital negative) and α4β7^+^ (α4-PE/β7-FITC) 293T cells. Two types of binding assays were done; namely a competition binding assay and a direct binding assay. In the first, α4β7 transfected 293T cells (1 × 10^5^ cells/ml) were incubated in the presence or absence of the Act-1 mAb (targets the α4β7 dimer) obtained from the NIH AIDS Research and Reference Reagent Program or the HP2/1 mAb (Beckman Coulter, France) which targets the α4 subunit only, for 15 min followed by the addition of the either IECs or IMCs for 25 min. Cells were stained as above. Binding to α4β7 was defined as the percentage difference between the median fluorescence intensity (MFI) of α4-PE and β7-FITC of α4β7-transfected cells without an inhibitory mAb or virus and the MFI α4β7-transfected cells with an inhibitory mAb or virus. The direct binding assay was carried out in a similar way except that the cell-virus complexes were stained for p24-FITC (KC57, Beckman Coulter) and the percentage p24 positive cells gated on single live 293T cells were compared between 293T untransfected (background) and α4β7 transfected 293T cells as a representation of bound virus.

### α4β7 mediated virus replication inhibition assay

ATRA-treated CD4+ T lymphocytes (25 μl of 4 × 10^6^ cells/ml) from a responder individual were either incubated with 25 μl of media (virus control), 10 μg/ml anti-CD4 monoclonal antibody (positive control), 275 pM inhibiting monoclonal antibody HP2/1 (targeting α4 subunit) or 2.2 pM Act-1 (targets α4β7) for an hour at 37°C at 5% CO2. Both HP2/1 and Act-1 mAb concentrations were optimised by titration in the α4β7-mediated virus capture inhibition assay, noting the concentration at which effectiveness of blocking viral replication was highest. 100 μl containing 25 TCID_50_ of IECs and IMCs were incubated for 2 h in triplicate with the cells in a 96-well plate, followed by two washes with fresh IL-2 media (5% IL-2 at 200 U/ml, 20% FBS and RPMI media) at 1,200 g for 5 min at 25°C. 100 µl of culture supernatant was harvested every 2 days without disturbing the cells for a period of 10 days with removed volume replaced with fresh IL-2 media. Collected supernatants were lysed using 1.25% Empigen/TBS solution and stored at 4°C before assessing p24 levels by ELISA. Percentage dependency on α4β7 was calculated as the percentage of difference in p24 between untreated samples (virus control) and p24 on samples treated with HP2/1 or Act-1 relative to untreated controls at the exponential growth phase of the latter. These single points were unique for each virus based on steepness of the gradient of the kinetic growth curve.

### Cytokine measurements in CVLs and plasma

CVLs (10 ml sterile saline) for cytokine measurements were collected, centrifuged and supernatants stored at −80°C. CVLs were not collected from menstruating participants. Blood was collected by venepuncture into acetate citrate dextran vacutainer tubes, plasma isolated and stored at −80°C. CVLs were pre-filtered by centrifugation using 0.2 μm cellulose acetate filters (Sigma, USA). The concentrations of 32 cytokines were measured using LINCO*plex* Human Cytokine and High Sensitivity Human Cytokine kits (LINCO Research, USA). Human Cytokine LINCO*plex* kits included Epidermal growth factor (EGF), eotaxin/CCL11, fractalkine/CX_3_CL1, G-CSF, IFN-α, IL-1α, IL-1Ra, IL-12p40, IL-15, IL-17, IFN-γ-induced protein (IP)-10/CXCL10, MCP-1/CCL2, MIP-1α/CCL3, MIP-1β/CCL4, RANTES/CCL5, sCD40L, soluble IL-2 receptor α (sIL-2Rα), transforming growth factor (TGF)-α and vascular endothelial growth factor (VEGF). Thirteen cytokines were measured in CVL and plasma using High Sensitivity LINCO*plex* kits: IL-1β, IL-2, IL-4, IL-5, IL-6, IL-7, IL-8/CXCL8, IL-10, IL-12p70, IL-13, GM-CSF, IFN-γ and TNF-α. The lower limit of detection of these kits ranged between 0.01 and 27.65 pg/ml for each of the cytokines. Data was collected using a Bio-Plex™ Suspension Array Reader (Bio-Rad Laboratories Inc^®^) and a 5 PL regression formula was used to calculate cytokine concentrations from the standard curves (BIO-Plex™ manager software version 4). IFN-α and MIP-3α were measured in CVLs using ELISA (R&D Systems). Cytokine concentrations below the assay lower limit of detection were reported as the mid-point between the lowest concentrations measured for each cytokine and zero.

### Statistical analysis

Paired data with only two groups were assessed by paired t test or Wilcoxon matched pairs signed rank test, and in groups with three or more sets by the repeated measures one way ANOVA with pairwise comparisons adjusted for multiple comparisons using Tukey’s method. Unpaired data were assessed for significance by the Mann–Whitney test, or the one way ANOVA and the Fisher exact test was used to determine the significance of categorical groupings. While Spearman’s correlation was used to assess linear correlations, correlations were adjusted for multiple comparisons using a false discovery rate (FDR) step down procedure (STATA version 12, StataCorp, College Station, TX, USA). A mixed linear model was fitted to assess significance in cases where there were multiple comparisons with repeated measures and were corrected for duration of infection. Observations of p < 0.05 were defined as significant. Statistics and graphs were done with GraphPad Prism 5 and STATA 12.

## Additional files

Additional file 1: α4β7-virus binding assays. (**A**) α4β7 expressed on transfected 293T cells (red) and not on the surface of untransfected 293T cells (black) confirmed by flow cytometry were used for direct binding and competition assays. (**B**) Infectious viruses represented by p24-FITC MFI bound to α4β7 transfected 293T cells shown in red but not to untransfected 293 cells shown in black, representative of 4 independent experiments. (**C**) Viral attachment of CAP88 T/F in the presence of HP2/1 and Act-1 was inhibited in the p24 direct binding assay (red and blue respectively), representative of 5 independent experiments. Additional file 2: IECs and IMCs show similar binding to and dependence on α4β7 for replication. IECs and IMCs of CAP210 T/F and CAP239 T/F bound to α4β7 at similar levels as defined by (**A**) p24 binding assay where the dashed line represents IMC binding and the solid line represents IEC binding and (**B**) the competition assay. SF162 IEC and IMC also bound similarly see Figure 1A. (**C**) All three viruses showed no differences between their IECs and respective IMCs in dependence on α4β7 for replication. All results are representative of three experiments with error bars representing SEM. No differences were significant by the paired t test. Additional file 3: Characterisation of a responder and non-responder to ATRA treatment. CD4 cells were treated with ATRA for 6 days and stained for α4 and β7. An example of a non-responder is shown in A and a responder in B with an increase in the percentage of cells with α4 and β7 co-expression. Additional file 4: Titration of α4β7 inhibitors for maximal inhibition of viral replication. (**A**) A decreasing shift in PE florescence relative to the experimental control (ATRA treated CD4 + T cells alone shown in black); is indicated in a dose-dependent manner between 0.0022-30 nM (i) HP2/1 and (ii) Act-1 concentrations (red and blue graduations respectively). All samples were gated on single live CD4 + T lymphocytes. (**B**) Change in median fluorescent intensity (MFI) with the 30 nM being the most saturating concentration for blocking of the integrin by both mAbs. Both A and B are representative of four repeated experiments, with the bars showing the mean and error bars, the SEM. (**C**) The same ATRA activated responders were infected with a T/F virus CAP88.2.00.17-5A and incubated with (i) HP2/1 and (ii) Act-1 between 0.0022-30 nM (red and blue graduations respectively) and monitored over 10 days by p24-ELISA. The virus control (no inhibitory mAb) is shown in black and the infectivity control shown in grey. The optimum concentration for viral inhibition by HP2/1 is 0.275 nM and Act-1 is 2.2 pM, while the viral replication is significantly upregulated at 30 nM in both cases. These results are representative of five independent experiments using 3 donors, with error bars indicating the SEM and points, the mean of three replicates. Additional file 5: α4β7 mediated virus capture inhibition assay for all 60 IECs. Virus growth kinetic curves of CAP8, 88, 177, 200, 206, 210, 225, 239, 244, 255 and 256 viruses done in triplicate; in the presence or absence of HP2/1 mAb (red), Act-1 mAb (blue) or CD4 mAb (grey) demonstrate their inhibitory effect on the replication of T/F, early and chronic clones. Data are representative of two independent experiments, with the curves representative of the mean p24 readings and error bars indicating the SEM. The longitudinal samples including a T/F from the 3 individuals included in Figure 2 are indicated by an asterisk. Additional file 6: Characteristics of IECs used in this study. Additional file 7: gp160 sequence alignment of matched T/F and acute virus pairs. Changes between the T/F and acute (2-6 month) viruses for 3 pairs (CAP88, CAP200, CAP206) are indicated. Important functional and structural regions of gp160 are highlighted. Predicted N-linked glycans are shown in CAPS and red. Additional file 8: The impact of HIV glycans on α4β7 reactivity. (**A**) Correlations between the glycan density, the length of V1/V2, V3, C3/V4, V5 and dependence on α4β7 (Act-1) for replication of 60 IEC. Blocks shaded in grey are those which were significant in a linear mixed model corrected for repeated measures. Beta coefficients and p values are shown below the respective blocks. (**B**) Frequency of predicted N-linked glycans in the conserved regions of gp120 relative to the percentage of viruses that showed high (green, n = 33) or low (blue, n = 27) dependency on α4β7. Significance was determined by the Fisher exact test where PNG234 (**p = 0.009) and PNG334 (**p = 0.006) were more frequent in viruses with high α4β7 dependence andPNG332 was more frequent in viruses with low α4β7 dependence(*p = 0.026). Additional file 9: Univariate correlations between α4β7 dependence for replication and cytokine levels in CVL and plasma at transmission.
