# Supplementary material for: South African HIV-1 subtype C transmitted variants with a specific V2 motif show higher dependence on α4β7 for replication
Source: Retrovirology. 2015 Jun 24;12:54. doi: 10.1186/s12977-015-0183-3 (PMC4479312; doi:10.1186/s12977-015-0183-3)
Supplement: Additional file 1: — α4β7-virus binding assays. (A) α4β7 expressed on transfected 293T cells (red) and not on the surface of untransfected 293T cells (black) confirmed by flow cytometry were used for direct binding and competition assays. (B) Infectious viruses represented by p24-FITC MFI bound to α4β7 transfected 293T cells shown in red but not to untransfected 293 cells shown in black, representative of 4 independent experiments. (C) Viral attachment of CAP88 T/F in the presence of HP2/1 and Act-1 was inhibited in the p24 direct binding assay (red and blue respectively), representative of 5 independent experiments. [file 12977_2015_183_MOESM1_ESM.pdf]

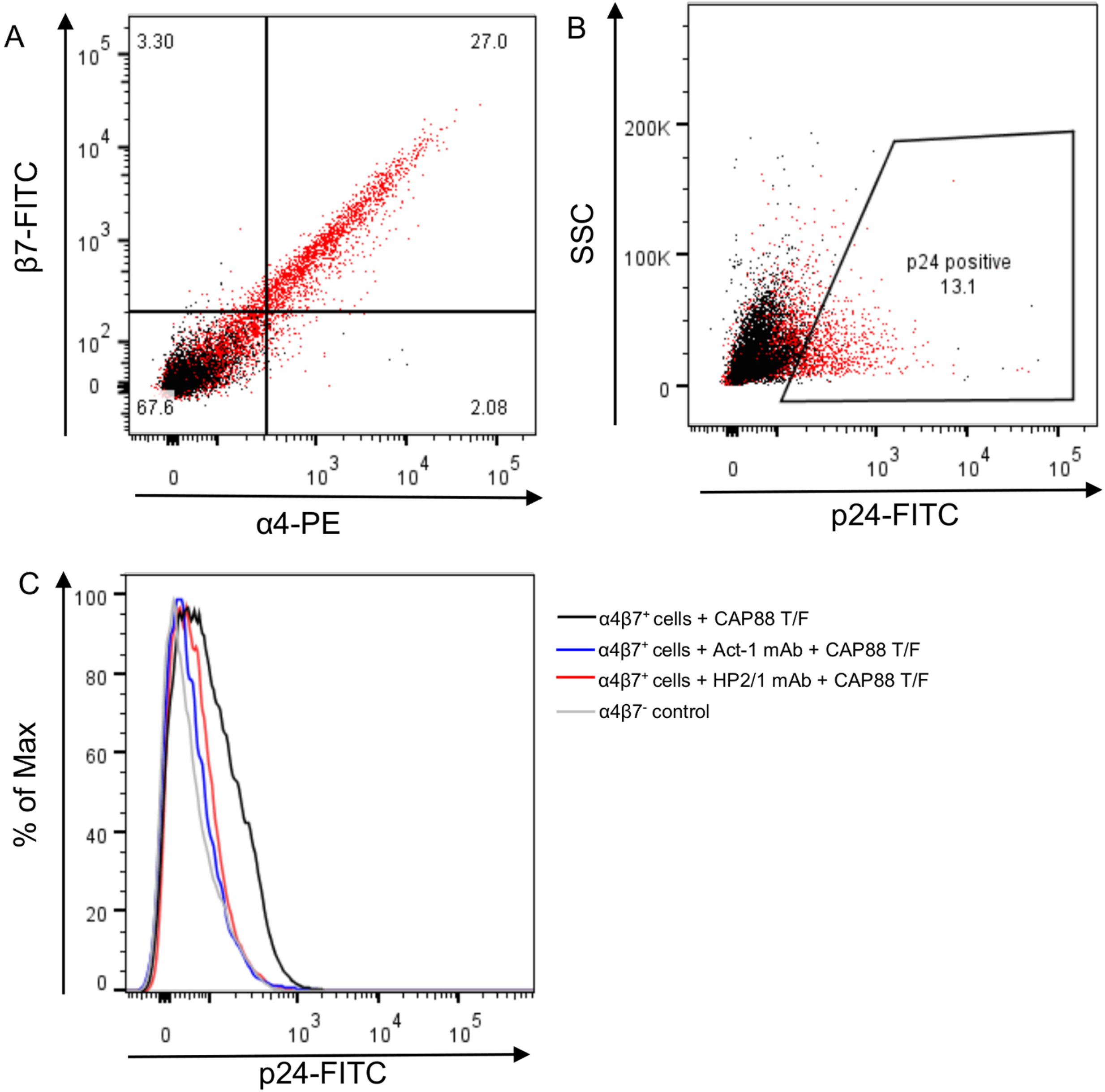

A
B
C
β7-FITC
α4-PE
3.30
27.0
67.6
2.08
SSC
p24-FITC
200K
100K
p24 positive
13.1
% of Max
α4β7⁺ cells + CAP88 T/F
α4β7⁺ cells + Act-1 mAb + CAP88 T/F
α4β7⁺ cells + HP2/1 mAb + CAP88 T/F
α4β7⁻ control
