# Supplementary figures and images for: South African HIV-1 subtype C transmitted variants with a specific V2 motif show higher dependence on α4β7 for replication
Source: Retrovirology. 2015 Jun 24;12:54. doi: 10.1186/s12977-015-0183-3 (PMC4479312; doi:10.1186/s12977-015-0183-3)

A

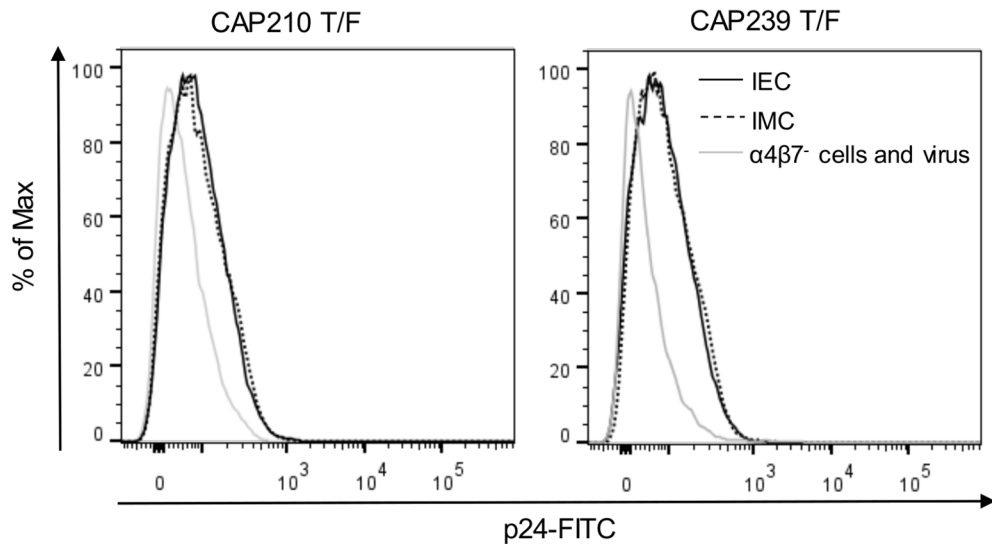

B

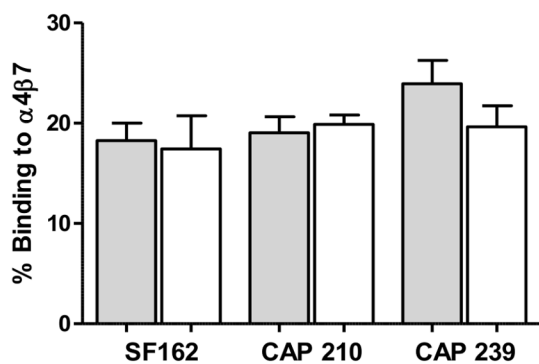

C

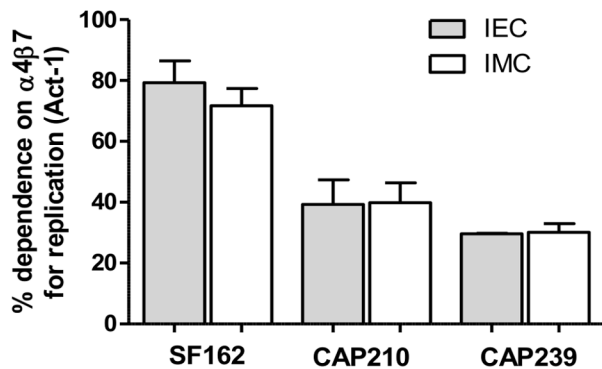

Supplement: Additional file 2: — IECs and IMCs show similar binding to and dependence on α4β7 for replication. IECs and IMCs of CAP210 T/F and CAP239 T/F bound to α4β7 at similar levels as defined by (A) p24 binding assay where the dashed line represents IMC binding and the solid line represents IEC binding and (B) the competition assay. SF162 IEC and IMC also bound similarly see Figure 1A. (C) All three viruses showed no differences between their IECs and respective IMCs in dependence on α4β7 for replication. All results are representative of three experiments with error bars representing SEM. No differences were significant by the paired t test. [file 12977_2015_183_MOESM2_ESM.pdf]

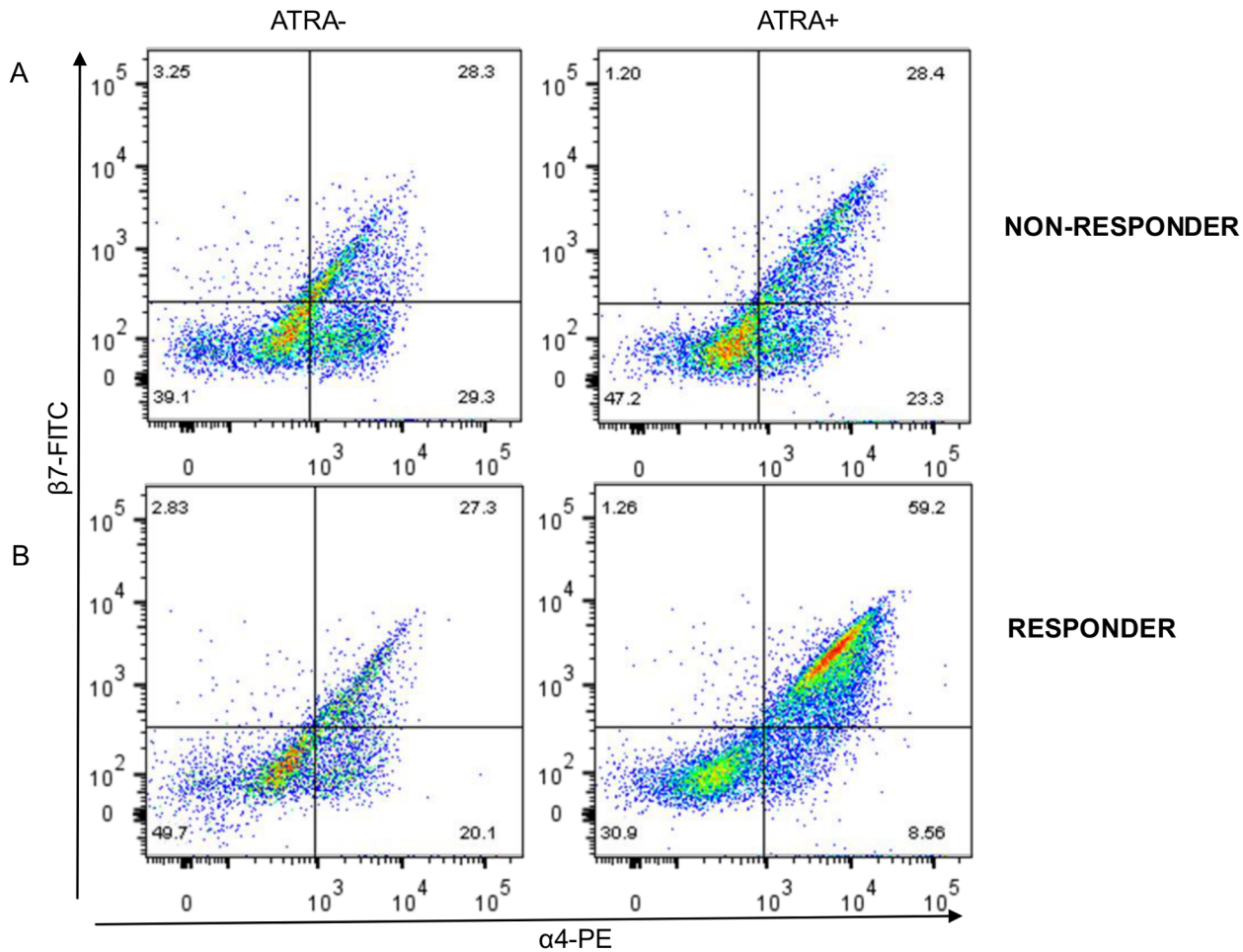

Supplement: Additional file 3: — Characterisation of a responder and non-responder to ATRA treatment. CD4 cells were treated with ATRA for 6 days and stained for α4 and β7. An example of a non-responder is shown in A and a responder in B with an increase in the percentage of cells with α4 and β7 co-expression. [file 12977_2015_183_MOESM3_ESM.pdf]

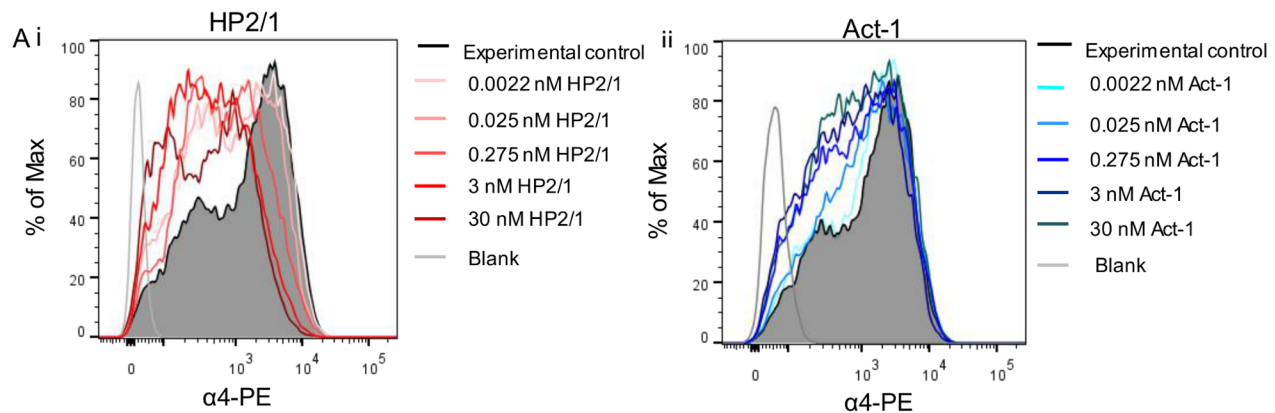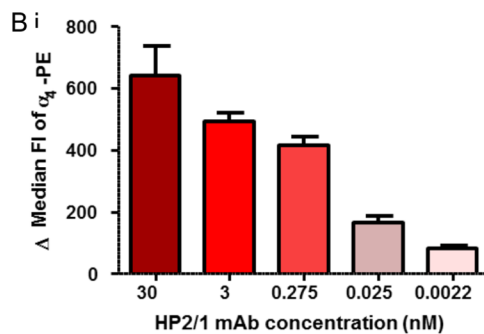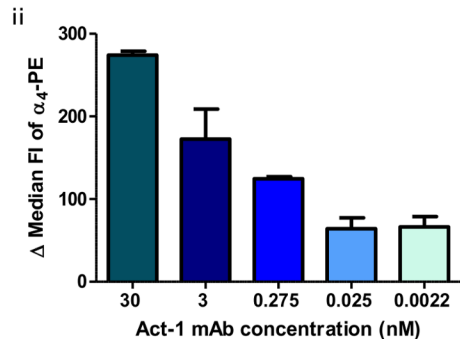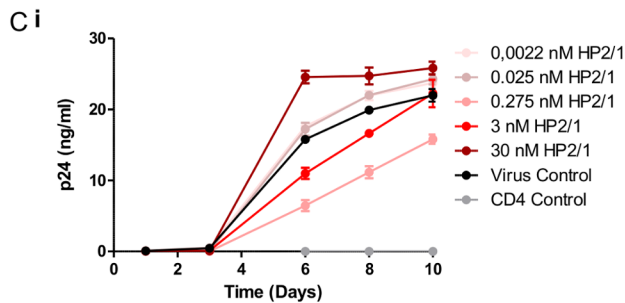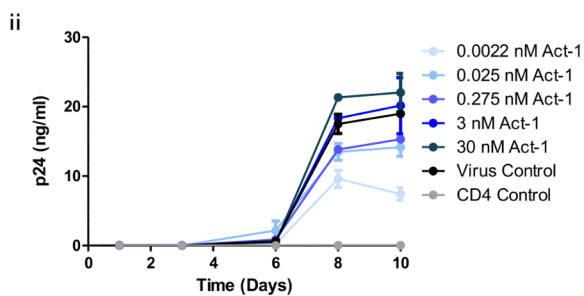

Supplement: Additional file 4: — Titration of α4β7 inhibitors for maximal inhibition of viral replication. (A) A decreasing shift in PE florescence relative to the experimental control (ATRA treated CD4 + T cells alone shown in black); is indicated in a dose-dependent manner between 0.0022-30 nM (i) HP2/1 and (ii) Act-1 concentrations (red and blue graduations respectively). All samples were gated on single live CD4 + T lymphocytes. (B) Change in median fluorescent intensity (MFI) with the 30 nM being the most saturating concentration for blocking of the integrin by both mAbs. Both A and B are representative of four repeated experiments, with the bars showing the mean and error bars, the SEM. (C) The same ATRA activated responders were infected with a T/F virus CAP88.2.00.17-5A and incubated with (i) HP2/1 and (ii) Act-1 between 0.0022-30 nM (red and blue graduations respectively) and monitored over 10 days by p24-ELISA. The virus control (no inhibitory mAb) is shown in black and the infectivity control shown in grey. The optimum concentration for viral inhibition by HP2/1 is 0.275 nM and Act-1 is 2.2 pM, while the viral replication is significantly upregulated at 30 nM in both cases. These results are representative of five independent experiments using 3 donors, with error bars indicating the SEM and points, the mean of three replicates. [file 12977_2015_183_MOESM4_ESM.pdf]

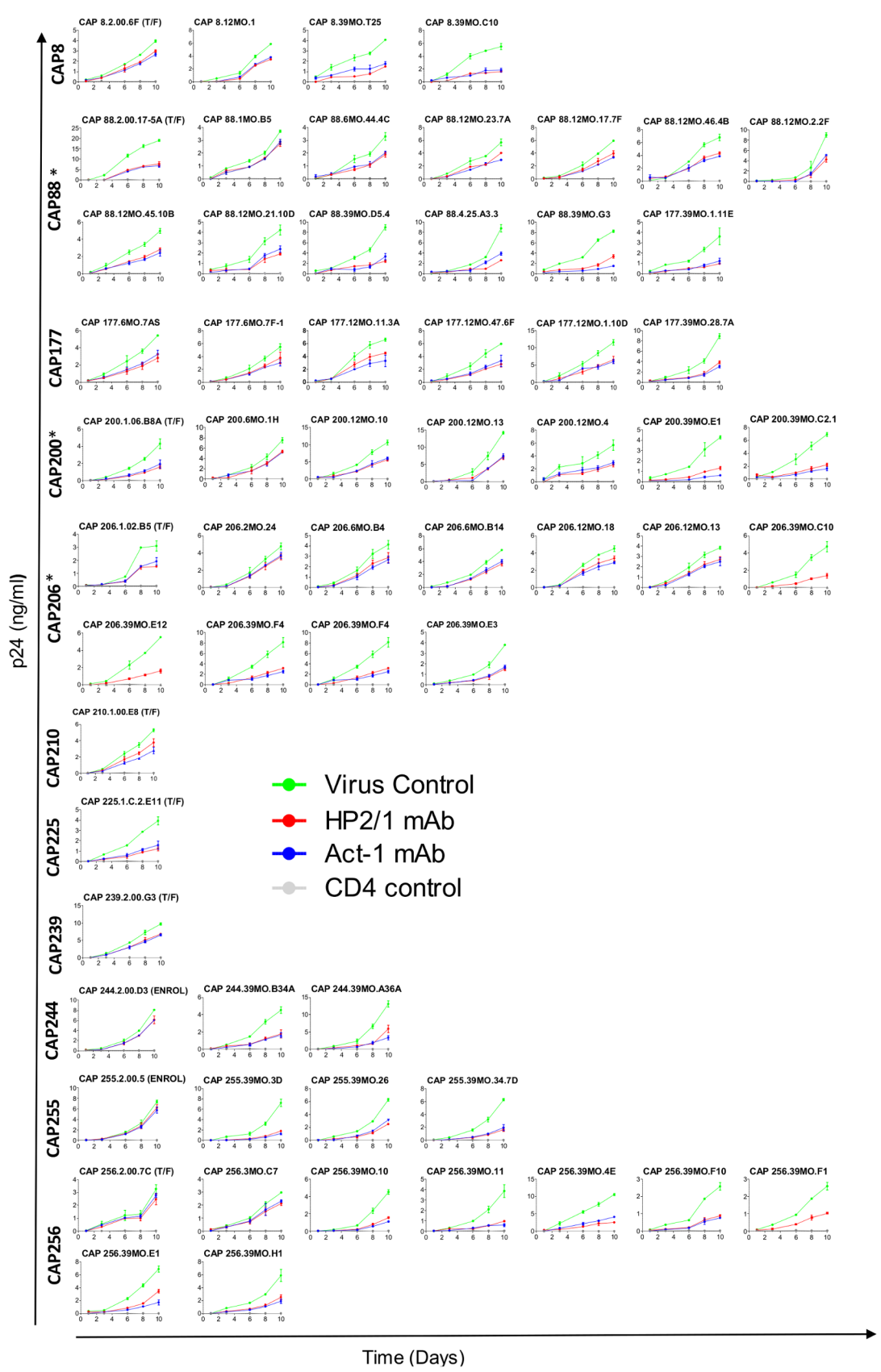

Supplement: Additional file 5: — α4β7 mediated virus capture inhibition assay for all 60 IECs. Virus growth kinetic curves of CAP8, 88, 177, 200, 206, 210, 225, 239, 244, 255 and 256 viruses done in triplicate; in the presence or absence of HP2/1 mAb (red), Act-1 mAb (blue) or CD4 mAb (grey) demonstrate their inhibitory effect on the replication of T/F, early and chronic clones. Data are representative of two independent experiments, with the curves representative of the mean p24 readings and error bars indicating the SEM. The longitudinal samples including a T/F from the 3 individuals included in Figure 2 are indicated by an asterisk. [file 12977_2015_183_MOESM5_ESM.pdf]

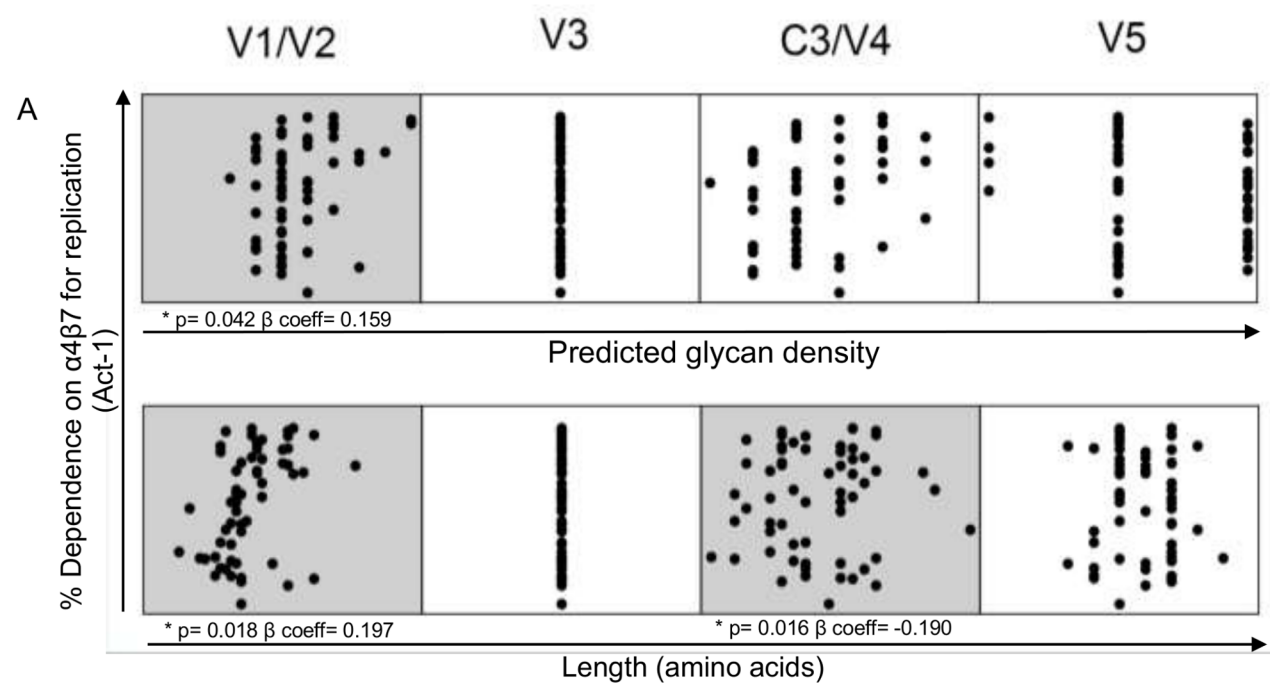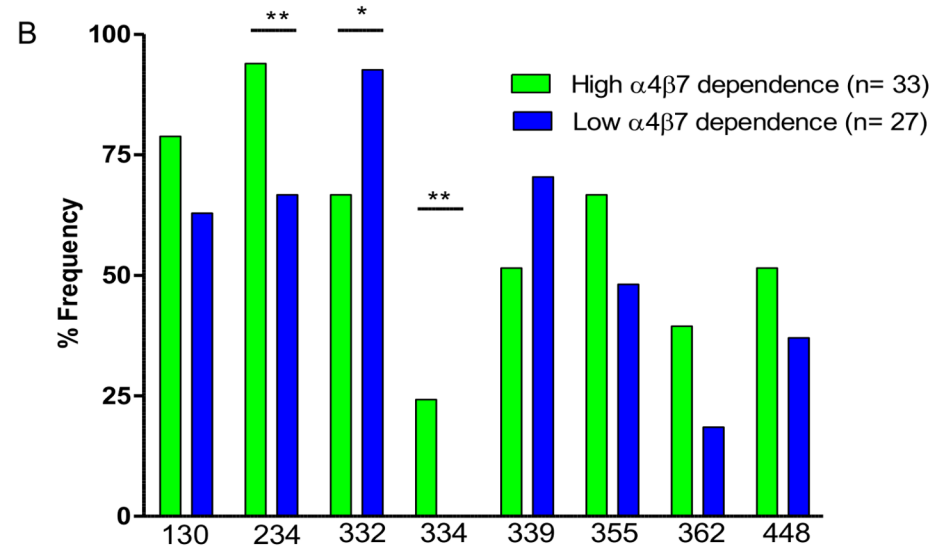

Supplement: Additional file 8: — The impact of HIV glycans on α4β7 reactivity. (A) Correlations between the glycan density, the length of V1/V2, V3, C3/V4, V5 and dependence on α4β7 (Act-1) for replication of 60 IEC. Blocks shaded in grey are those which were significant in a linear mixed model corrected for repeated measures. Beta coefficients and p values are shown below the respective blocks. (B) Frequency of predicted N-linked glycans in the conserved regions of gp120 relative to the percentage of viruses that showed high (green, n = 33) or low (blue, n = 27) dependency on α4β7. Significance was determined by the Fisher exact test where PNG234 (**p = 0.009) and PNG334 (**p = 0.006) were more frequent in viruses with high α4β7 dependence andPNG332 was more frequent in viruses with low α4β7 dependence(*p = 0.026). [file 12977_2015_183_MOESM8_ESM.pdf]
