# Supplementary material for: South African HIV-1 subtype C transmitted variants with a specific V2 motif show higher dependence on α4β7 for replication
Source: Retrovirology. 2015 Jun 24;12:54. doi: 10.1186/s12977-015-0183-3 (PMC4479312; doi:10.1186/s12977-015-0183-3)
Supplement: Additional file 7: — gp160 sequence alignment of matched T/F and acute virus pairs. Changes between the T/F and acute (2-6 month) viruses for 3 pairs (CAP88, CAP200, CAP206) are indicated. Important functional and structural regions of gp160 are highlighted. Predicted N-linked glycans are shown in CAPS and red. [file 12977_2015_183_MOESM7_ESM.pdf]

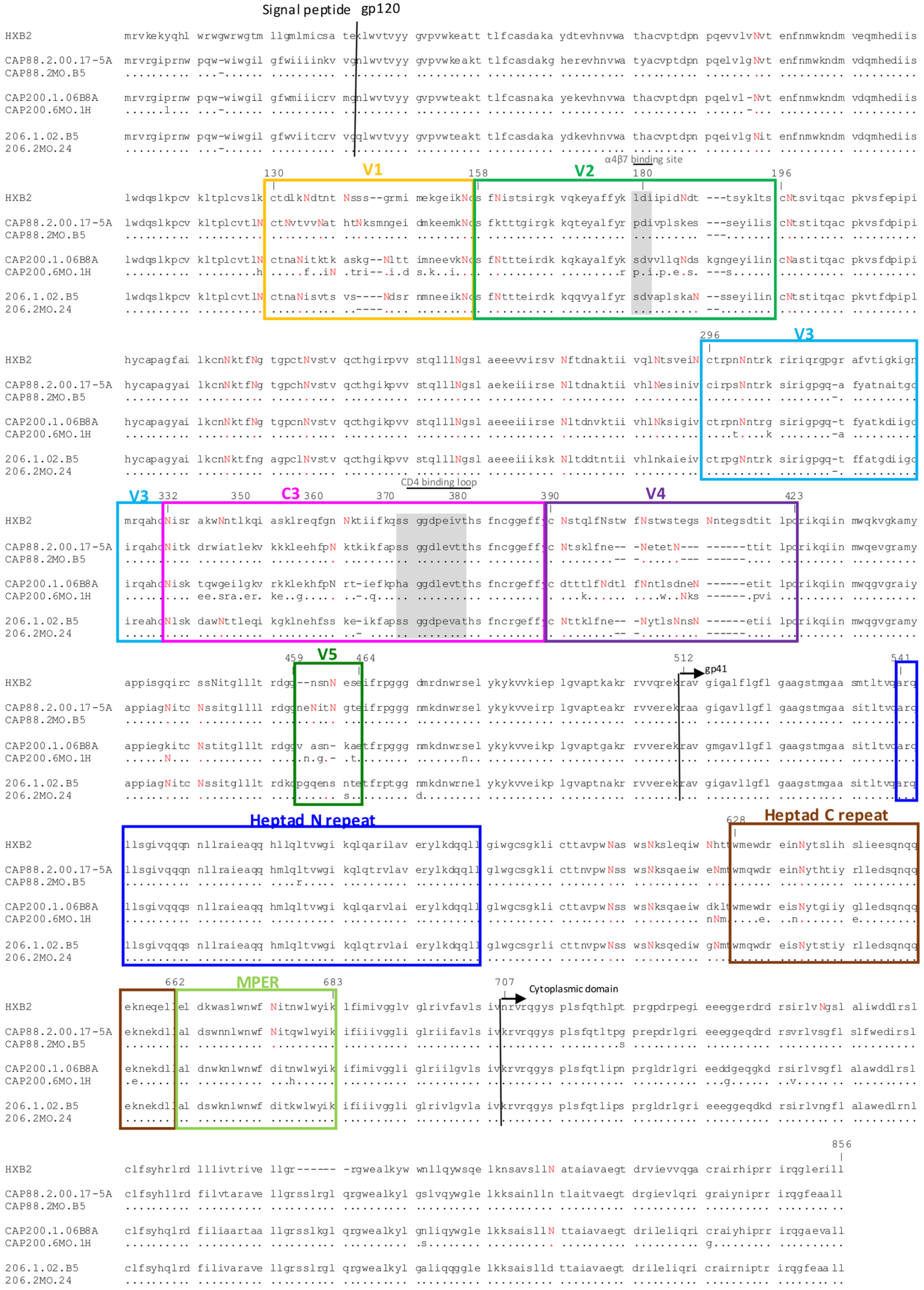

Signal peptide gp120
HXB2 mrvkekyqhl wrwgwrwgtm llgmlmicsa teklwvtvyy gvpvwkeatt tlfcasdaka ydtevhnvwa thacvptdpn pqevvlvNvt enfnmwkndm veqmhediis
CAP88.2.00.17-5A mrvrgiprnw pqw-wiwgil gfwiiiinkv vgnlwvtvyy gvpvwkeakt tlfcasdakg herevhnvwa tyacvptdpn pqelvlgNvt enfnmwkndm vdqmhediis
CAP88.2MO.B5
CAP200.1.06B8A mrvrgiprnw pqw-wiwgil gfwmiiicrv mgnlwvtvyy gvpvwteakt tlfcasnaka yekevhnvwa thacvptdpn pqelvl-Nvt enfnmwkndm vdqmhediis
CAP200.6MO.1H
206.1.02.B5 mrvrgiprnw pqw-wiwgil gfwviitcrv vqqlwvtvyy gvpvwteakt tlfcasdaka ydkevhnvwa thacvptdpn pqeivlgNit enfnmwkndm vdqmhediis
206.2MO.24
α4β7 binding site
130 V1 158 V2 180 196
HXB2 lwdqslkpcv kltplcvslk ctdlkNdtnt Nsss--grmi mekgeikNcs fNistsirgk vqkeyaffyk ldiipidNdt ---tsyklts cNtsvitqac pkvsfepipi
CAP88.2.00.17-5A lwdqslkpcv kltplcvtlN ctNvtvvNat htNksmngei dmkeemkNcs fktttgirgk kqteyalfyr pdivplskes ---seyilis cNtstitqac pkvsfdpipi
CAP88.2MO.B5
CAP200.1.06B8A lwdqslkpcv kltplcvtlN ctnaNitktk askg--Nltt imneevkNcs fNttteirdk kqkayalfyk sdvvllqNds kgngeyilin cNastitqac pkvsfdpipi
CAP200.6MO.1H h ....f..iN .tri--.i.d s.k..i... r p.i.p.e.s. ---s......
206.1.02.B5 lwdqslkpcv kltplcvtlN ctnaNisvts vs----Ndsr mmneeikNcs fNttteirdk kqqvyalfyr sdvaplskaN --sseyilin cNtstitqac pkvtfdpipl
206.2MO.24
296 V3
HXB2 hycapagfai lkcnNktfNg tgpctNvstv qcthgirpvv stqlllNgsl aeeevvirsv Nftdnaktii vqlNtsveiN ctrpnNntrk ririqrgpgr afvtigkign
CAP88.2.00.17-5A hycapagyai lkcnNktfNg tgpchNvstv qcthgikpvv stqlllNgsl aekeiiirse Nltdnaktii vhlNesiniv cirpsNntrk sirigpgq-a fyatnaitgd
CAP88.2MO.B5
CAP200.1.06B8A hycapagyai lkcnNktfNg tgpcnNvstv qcthgikpvv stqlllNgsl aeeeiiirse Nltdnvktii vhlNksigiv ctrpnNntrg sirigpgq-t fyatkdiigd
CAP200.6MO.1H ....t....k ....-a
206.1.02.B5 hycapagyai lkcnNktfng agpclNvstv qcthgikpvv stqlllNgsl aeeeiiiksk Nltddtntii vhlnkaieiv ctrpgNntrk sirigpgq-t ffatgdiigd
206.2MO.24
CD4 binding loop
V3 332 350 C3 360 370 380 390 V4 423
HXB2 mrqahcNisr akwNntlkqi asklreqfgn Nktiifkqss ggdpeivths fncggeffyc NstqlfNstw fNstwstegs Nntegsdtit lpcrikqiin mwqkvgkamy
CAP88.2.00.17-5A irqahcNitk drwiatlekv kkkleehfpN ktkikfapss ggdlevtths fncggeffyc Ntsklfne-- -NetetN--- ------ttit lpcrikqiin mwqevgramy
CAP88.2MO.B5
CAP200.1.06B8A irqahcNisk tqwgeilgkv rkklekhfpN rt-iefkpha ggdlevtths fncrgeffyc dtttlfNdtl fNntlsdneN ------etit lpcrikqiin mwqgvgraiy
CAP200.6MO.1H ee.sra.er. ke..g.... ..-.q. ...k. ....w..Nks ------.pvi
206.1.02.B5 ireahcNlsk dawNttleqi kgklnehfss ke-ikfapss ggdpevaths fncrgeffyc Nttklfne-- -NytlsNnsN ------etii lpcrikqiin mwqgvgramy
206.2MO.24
459 V5 464 512 gp41 541
HXB2 appisgqirc ssNitglllt rdgg--nsnN eseifrpggg dmrdnwrsel ykykvvkiep lgvaptkakr rvvqrekrav gigalflgfl gaagstmgaa smtltvqarq
CAP88.2.00.17-5A appiagNitc Nssitgllll rdggneNitN gteifrpggg nmkdnwrsel ykykvveirp lgvapteakr rvverekraa gigavllgfl gaagstmgaa sitltvqarq
CAP88.2MO.B5
CAP200.1.06B8A appiegkitc Nstitglllt rdggv asn- kaetfrpggg nmkdnwrsel ykykvveikp lgvaptgakr rvverekrav gmgavllgfl gaagstmgaa sitltvqarq
CAP200.6MO.1H ......N... .n.g.- .t. ....n..
206.1.02.B5 appiagNitc Nssitgllt rdkqpgqens ntetfrptgg nmkdnwrnel ykykvveikp lgvaptnakr rvverekrav gigavllgfl gaagstmgaa sitltvqarq
206.2MO.24 s. d.
Heptad N repeat 628 Heptad C repeat
HXB2 llsgivqqqn nllraieaqq hllqltvwgi kqlqarilav erylkdqqll giwgcsgkli cttavpwNas wsNksleqiw Nhttwmewdr einNytslih slieesqnqq
CAP88.2.00.17-5A llsgivqqqn nllraieaqq hmlqltvwgi kqlqtrvlav erylkdqqll glwgcsgkli cttnvpwNss wsNksqaeiw eNmtwmqwdr einNythtiy rlledsqnqq
CAP88.2MO.B5 ....r.....
CAP200.1.06B8A llsgivqqqs nllraieaqq hmlqltvwgi kqlqarvlai erylkdqqll glwgcsgkli cttavpwNss wsNksqaeiw dkltwmewdr eisNytgiiy glledsqnqq
CAP200.6MO.1H nNm.....e. ..n....... e.........
206.1.02.B5 llsgivqqqs nllraieaqq hmlqltvwgi kqlqtrvlai erylkdqqll glwgcsgrli cttnvpwNss wsNksqediw gNmtwmqwdr eisNytstiy rlledsqnqq
206.2MO.24
662 MPER 683 707 Cytoplasmic domain
HXB2 ekneqell el dkwaslwnwf Nitnwlwyik lfimivgglv glrivfavls ivnrvrqgys plsfqthlpt prgpdrpegi eeeggerdrd rsirlvNgsl aliwddlrsl
CAP88.2.00.17-5A eknekdll al dswnnlwnwf Nitqwlwyik ifiiivggli glriifavls ivkrvrqgys plsfqtltpg prepdrlgri eeeggeqdrd rsvrlvsgfl slfwedirsl
CAP88.2MO.B5 s
CAP200.1.06B8A eknekdll al dnwknlwnwf ditnwlwyik ifimivggli glriilgvls ivkrvrqgys plsfqtlipn prgldrlgri eeddgeqgkd rsirlvsgfl alawddlrsl
CAP200.6MO.1H .e..... ...h..... ...g...... ..v.......
206.1.02.B5 eknekdll al dswknlwnwf ditkwlwyik ifiiivggli glrivlgvla ivkrvrqgys plsfqtlips prgldrlgri eeeggeqdkd rsirlvngfl alawedlrnl
206.2MO.24
856
HXB2 clfsyhrlrd lllivtrive llgr------ -rgwealkyw wnllqywsqe lknsavsllN ataiavaegt drvievvqga crairhiprr irqglerill
CAP88.2.00.17-5A clfsyhllrd filvtarave llgrsslrgl qrgwealkyl gslvqywgle lkksainlln tlaitvaegt drgievlqri graiynipri irqgfeaall
CAP88.2MO.B5
CAP200.1.06B8A clfsyhqlrd filiaartaa llgrsslkgl qrgwealkyl gnliqywgle lkksaisllN ttaiavaegt drileliqri craiyhiprr irqgaevall
CAP200.6MO.1H .s........ g.........
206.1.02.B5 clfsyhqlrd filivarave llgrsslrgl qrgwealkyl galiqqggle lkksaislld ttaiavaegt drileliqri crairniptr irqgfeaa ll
206.2MO.24
